# Quality indicators for ICU: ISCCM guidelines for ICUs in India

**Published:** 2009

**Authors:** B. Ray, D. P. Samaddar, S. K. Todi, N. Ramakrishnan, George John, Suresh Ramasubban

**Affiliations:** **From:** Indian Society of Critical Care Medicine Instituted Task Force, India

## Foreword by Chair

Intensive care has had a phenomenal growth since its inception during the Copenhagen Poliomyelitis outbreak in 1952. Few specialities have grown with that much pace as that of Intensive Care, in such a short period. True, it is a ‘capital intensive’ care, but it saves lives, which otherwise would not have been possible, and even contributes, with precision, to perception of the future course of the disease and therefore to instituting remedial measures well ahead of time; as these patients require critical care therapies. Target-oriented therapies and bundles are becoming the preferred modalities to improve outcomes and there are definite indications that such therapies are helpful. Intensive therapy outcomes have been constantly improving, notwithstanding the variations in deployment of processes, resources, drugs, consumables and techniques in different ICUs. While disease outcomes are relatively easy to appreciate and account for, intensive care outcomes are not so easy to appreciate and account for, because of the very nature of the units and the way we practice intensive care, particularly in our country with a large number of open, very few semi-closed and even fewer closed units. In order to develop the right kind of unit and practice optimum therapies to provide best quality treatment to our critically ill patients, we need to develop appropriate key performance indicators, which reflect the aspirations of patients, relatives and intensivists.

Developing key performance indicators and monitoring, auditing and improving those parameters is a dynamic process which requires standardization, improvement and innovation – the three arms of any improvement process in industry or service scenario. While standardization means ‘removing the outliers,’ i.e. reducing the standard deviation, improvement denotes gradual bettering of a parameter from the previous level with a degree of irreversible consistency. Innovation is, however, sporadic and often requires a thinking cap, which while maintaining the speed of standardization and improvement, quickly takes the parameters to a new level. In Total Quality Management (TQM) parlance, the first two are a part or product of daily management and the last one is a part or product of policy management. While standardization and improvement come with all-round participation in the unit, the innovation comes from a particular individual or a section of the people connected with the unit.

Small improvements through small group activities (SGAs), previously known as Quality Circles, are central to any improvement in a unit and bring about pride and involvement amongst the staff in ICU. While isolated improvement activities are important to engage members to start with, institutionalizing these activities is the ultimate goal of the unit for, only that will ensure a complete irreversibility of the process. The latter is possible if the problems are constantly identified in the process/procedure and improvement initiatives are taken to address those. Striving for results is extremely important and for that the team needs to identify and take care of the ‘vital few’ problems leaving the ‘trivial many;’ something like ‘triaging’ in mass casualty parlance.

Co-relating the improvement of the process/outcome parameter with the improvement activities is important; if it does not match, then either one has not chosen the parameter properly or the parameter needs further development in the form of precision and complexity or the ‘vital few’ problems have not been properly identified. A constant engagement with the improvement process is necessary on the part of the team. The parameter needs to be developed, validated and revalidated in the same unit and in different units among the similar and dissimilar case mix before it is finally accepted.

## Members' Details

**Table d32e119:** 

Dr B Ray:	General Manager (Medical Services) Tata Main Hospital, Jamshedpur
	drbray@tatasteel.com;
	09234510648
Dr D P Samaddar:	HOD Anaesthesiology and Critical Care, Tata Main Hospital, Jamshedpur
	drdpsamaddar@tatasteel.com;
	9234551849
Dr S K Todi:	Head of Medicine and Critical Care, AMRI, Kolkata
	subhashtodi@vsnl.com;
	09831202040
Dr George John:	Professor of Medicine, Head division of Critical Care, CMC, Vellore
	yokavi@yahoo.com; 09443626986
Dr N Ramakrishnan:	Director, Critical Care Services, Senior Consultant in Critical Care and Sleep Medicine, Apollo Hospital, Chennai
	icudoctor@gmail.com;
	09840855115
Dr S Ramasubban:	Director Critical Care, Apollo Gleaneagles Hospital, Kolkata
	drsuresh@hotmail.com;
	09831740837

## Preface

**Background:** Efficiency of any healthcare unit is judged by its quality indicators. However in our country monitoring the outcome through quality indicators is not yet institutionalized because of many reasons including the fact that majority of ICUs in India are being run as open or semi closed units, with unaccountable custodians. Dependency on the key performance indicators practiced by the developed countries, therefore, becomes inevitable wherever some degree of total quality management system is being adhered to. It is generally seen that a few of the hospitals in India attempt to evolve their own parameters either taking ideas from the “established parameters” or from their experience in Indian hospitals. Some of the parameters, when pursued year after year, do not express or reflect the aspirations of the intensivists. Selecting definitive and sensitive quality indicators and forming a data base at national level, is therefore required. The executive committee of the Indian Society of Critical Care Medicine (ISCCM), took a decision in the year 2008 to evolve Quality Indicators for ICUs in India and a task force was constituted under the convener ship of Dr B Ray to give its report.

**Objective:** The primary objective is to select suitable quality indicators for Indian intensive care units (ICUs). Development of national data base and meaningful utilization of this data base is the final objective.

**Parameters:** Common performance parameters (nominators) along with certain basic parameters (denominators) have been selected to find out quality indicators. Each indicator has been explained for ease of understanding and uniformity of practice. Based on the selected parameters, the Dashboard has been developed to monitor the data.

**Dashboard:** Dashboard includes the selected parameters which would be made available to participating institutions to report to a main body at pre-decided intervals.

**Caution and limitations:** Very common parameters have been selected in this report. Acceptability and utility of these parameters in the Indian scenario will have to be assessed over a period of time. Accordingly, parameters will be modified and may be a few parameters have to be even discontinued if those parameters do not reflect the outcome directly or indirectly.

**Future steps:** Addition and deletion of parameters, as per need, would be considered in future. This will be done in phases after proper evaluation of monitored parameters. National data base generated by this exercise can be released for public reporting at a later date. Institutions will also be in a position to compare their performance with the national data base.

**Approach by an Intensive Care Unit:** These should be the guidelines and by no means a complete or closed list. Once the parameters are put in place, and monitored and audited at predetermined intervals, one would surely find some improvement in the key performance indicators (KPIs); but by no standards should that alone be construed as a successful exercise. The approach should be to minimize standard deviation (prevent “spikes” on either direction) while improving the KPIs. It will be clearly appreciated that the whole unit's involvement is essential to identify the bottlenecks in the process or functional areas of any parameter and take remedial action through small group activities (SGAs) and self initiated projects (SIPs). One would see a lot of Plan-Do-Check-Act (PDCAs) on the way to evolution of a parameter.

## Main Report

**Table d32e243:** 

Index		Page no
1.	Background (Introduction):	175
2.	Gathering the Evidence:	175
3.	Units	175
4.	Objective	176
5.	Parameters	176
6.	Definition of Parameters	176
7.	Dashboard	183
8.	Limitations	183
9.	Future Course of Action	183
10.	Recommendations	184
11.	List of Symbols	184
12.	Acknowledgments	185
13.	References (additional)	185
14.	Annexure	185
14.1.	Quality Indicators in Critical Care: An Overview	185
14.2.	Quality Indicators in Critical Care: Patient Safety	192
14.3.	Quality Indicators in Critical Care: Personnel Development	194
14.4.	Quality Indicators for ICU: Process Parameters	196
14.5.	Quality Indicators in Critical Care: Outcome Parameters	200
14.6.	Quality Indicators: Infection Control	203

## 1. Background

Quality orientation is an integral part of patient care. The best possible care at the institutional level is not considered adequate in the present competitive environment. It should be visible, appreciated and comparable. Total Quality Management therefore, is essential to judge the appropriateness and effectiveness of medical care. Quality of service offered, result of intervention and treatment, undesirable outcomes, and other managerial and treatment related processes can be analyzed to define the scope of improvement. Quality indicators help in achieving these objectives. Healthcare is becoming transparent and customer-focused. Patients and their relatives have the right to know the standard of care and its cost.

It is therefore becoming more and more mandatory for an institution to monitor quality indicators/parameters, and compare their performance level with the national standard or international bench marks. It gives the individual institution an opportunity to improve its quality of care through standardization of processes, procedures and treatment protocols. Unfortunately, due to a variety of reasons, performance levels are not monitored in India and therefore a national data base does not exist for a meaningful comparison. Dependency on an international data base, even if not logical for Indian scenario, becomes inevitable in our strategic design and planning of the service.

In 2008, the Indian Society of Critical Care Medicine (ISCCM) had taken the initiative, at its executive body meeting, to identify quality indicators for ICUs in India, which will help ICUs in India judge their performance levels and compare them with the national data base.

## 2. Gathering Evidence

**Table d32e405:** 

*Annexure:*	
**1** Quality indicators in Critical	Dr B Ray,
Care: An overview	Dr D P Samaddar
**2** Patient safety	Dr S K Todi
**3** Personnel Development	Dr Suresh
**4** Quality of Processes	Dr George John,
	Dr N Ramakrishnan
**5** Outcome Parameters	Dr George John,
	Dr N Ramakrishnan
**6** Infection Control	Dr D P Samaddar

## 3. Units

This report focuses on adult mixed intensive care units, references have also been given (except Neonatal ICU) wherever possible, to benchmark other specified units).

Abbreviations used for different specialized units are given in Table 1.

**Table 1 T0001:** 

Unit	Abbreviation
Burn	BCU
Coronary	CCU
Surgical cardiothoracic	SCU
Medical	MICU
Medical/surgical, major, teaching	M-S ICU major teaching
Medical/surgical, all others	M- S ICU
Pediatric medical/surgical	PICU
Neurological	Neuro (Med) ICU
Neurosurgical	Neuro (Surg) ICU
Surgical	SICU
Trauma	TICU

## 4. Objective

Select very common parameters mainly focusing on the *Outcome* (mortality and morbidity), *process, infection, communication, human resource and safety*.Generate national data base for comparison with international bench marks and provide data to participating institutions at national level for comparison with national data base.

## 5. Parameters

Based on the objective of this report, common parameters with their international benchmarks have been selected to address different aspects of patient care, operational issues and human resource development. Certain basic data, which as such do not reflect patient care, but when used as denominators to the selected parameters, make the parameter more sensitive and meaningful. Examples of these denominators are: number of admissions, total patient days in the unit (occupancy), ventilatory days, central venous and arterial line days, urinary catheter days etc. In order to avoid confusion and ambiguity of interpretation, it is essential that purpose and usefulness of selected parameters must be understood by the care providers. All the selected parameters, therefore, are described under certain sub headings as given in the Table 2, along with explanations.

**Table 2 T0002:** 

Indicator	Explanation
Description	What does the parameter mean
Rationality	Why should it be monitored
Formula for calculation	How it should be calculated
Patient population	For whom the parameter is collected
Source of data	From where the input will be collected
Type of parameter	Linkage of parameter with the type of quality
Bench mark	Common national or international standard
References	Literature back up for the bench mark and background information for the selected parameter

## 6. Definition of Parameters

### 6.1 Mortality

#### 6.1.1 Standardized Mortality Rate(SMR)

**Table d32e630:** 

Indicator	Standardized mortality rate (SMR) or risk adjusted mortality rate
Description	Mortality rates are not often the indicators of performance even if those are often referred to. However, mortality rate related to prior prediction is a sensitive indicator for comparison. SMR allows comparison of actual performance of the institution with predicted performance, based on the average mortality as expressed by national or international data.

Rationality	Risk of death varies with severity of disease state, age, and co- morbid conditions. Crude mortality (overall mortality), therefore, is not a sensitive indicator. On the basis of influencing factors, SMR obviates limitation of crude mortality as data from a large pool of patients with similar diagnoses and risk factors are analyzed to get expected mortality for that group of patients. Data can be obtained from national records or international records. Mortality rate can be obtained from predictive models such as APACHE, SAPS, MPM etc.[2] The SMR is a very useful parameter, often used to compare outcomes in two or more groups under study. It also gives an opportunity to individual ICUs for improving the processes and techniques.

Formula for calculation[1]	Risk-adjusted Mortality 1
	= (Observed Rate/Risk-adjusted expected Rate) X100
	Observed rate = Actual death in ICU/institution.
	Risk adjusted expected rate = Predicted death rate by predictive Model

Interpretation[1]	**Equal to 100** – hospital's mortality rate and the expected average rate are the same **>100** – hospitals' mortality rate is higher than the expected average mortality rate **<100** – hospitals mortality rate is lower than the expected average mortality rate Higher SMR does not necessarily mean that the hospital is unsafe, as this is a snapshot method and simultaneous assessment of other quality indicators must be done to draw a logical conclusion. Single parameter-based judgment on performance level is not advocated.[2]

Patient population	All patients admitted to critical care units of different types

Source of data	Hospital record for the observed mortality (numerator)

Type of parameter	Outcome

Bench mark	If the 95% confidence interval of the SMR includes 1, the performance is considered average. If the 95% CI *does not include 1, SMRs less than 1 and more than 1 are considered to show good and poor performances respectively.[3]

References	Available at: http://www.mayoclinic.org/quality/adjustedmortality.html Available at: http://www.qhc.on.ca/body.cfmid=565 Afessa B, Gajic O, Keegan MT. Keegan severity of illness and organ failure assessment in adult intensive care units. Crit Care Clin 2007;23:639-58.

### 6.2 Morbidity Parameters

#### 6.2.1. Iatrogenic Pneumothorax

**Table d32e773:** 

Indicator	Iatrogenic Pneumothorax
Description	Procedure related pneumothorax

Rationality	Associated mortality and morbidity, prolonged stay, cost implications

Formula for calculation	(Number of pneumothorax / Number of cases) X 1000

Patient population	Intensive care

Source of data	Hospital record

Type of parameter	Morbidity, safety

Bench mark	0.83per1000cases[1] 5% (interstitial emphysema/ pneumothorax /pneumomediastinum /subcutaneous emphysema)[2]

References	1. AHRQ national average. Sharp health care. Malcolm Baldrige National Quality Award application, 2007.
	2. Delgado MC, Pericas LC, Moreno JR, *et al.* Quality indicators in critically ill patients. SEMICYUC work groups. 1^st^ ed. May 2005. ISBN 6095974.

#### 6.2.2 Incidence of Acute Renal Failure in Noncoronary ICU

##### Noncoronary ICU

**Table d32e866:** 

Indicator	Incidence of severe Acute Renal Failure in noncoronary ICU
Description	Denovo acute renal failure requiring renal replacement therapy or when urine output is < 200 mL in 12 h and/or marked azotemia defined as a BUN level > 84 mg/dL) during patient's ICU stay.[1]

Rationality	Renal failure increases possibility of death (60.3%) notwithstanding whether renal replacement therapy has been initiated.[1,2] Even a modest increase in the serum creatinine level (0.3 to 0.4 mg per deciliter [26.5 to 35.4 ímol per liter]) increases risk of death by 70% when compared to normal creatinine levels.

Formula for calculation	Number developed severe renal failure/Number managed in ICU X 100

Patient population	Nominator: Severe renal failure (GFR < 10 ml/min.)[4] developing in ICU (excluding chronic renal failure patients.) Denominator: Patient managed in ICU in a given time frame

Source of data	ICU record

Type of parameter	Outcome parameter

Bench mark	Severe ARF 5.7%[1] 10% patients develop ARF (including Severe ARF)[4]

References	Uchino S, Kellum JA, Bellomo R, Doig GS, Morimatsu H, Morgera S, *et al*. Acute renal failure in critically ill patients: a multinational, multicenter study. JAMA 2005;294:813-8. Venkataraman R, Kellum JA. Prevention of Acute Renal Failure. Chest 2007;131:300-8. Chertow GM, Burdick E, Honour M, Bonventre JW, Bates DW. Acute kidney in jury, mortality, length of stay, and costs in hospitalized patients. J Am Soc Nephrol 2005;16:3365-70. Delgado MC, Pericas LC, Moreno JR, *et al*. Quality indicators in critically ill patients. SEMICYUC work groups. 1st ed. May ISBN; 2005. p. 609-5974.

#### 6.2.3. Decubitus (Pressure) Ulcer:

**Table d32e975:** 

Indicator	Decubitus (Pressure) ulcer
Description	Decubitus ulcer and pressure sore are synonyms. Decubitus is derived from the Latin word ***decumbere***, means “to lie down”. Since pressure sore can develop from other positions, it is called “Pressure sore”. Prolonged uninterrupted pressure over bony prominences causes necrosis and ulceration. Depending upon tissue damage, ulcers are classified into four stages. Stage 1 indicates superficial color change, Stage 2 represents partial thickness skin loss, Stage 3: full thickness skin loss, and Stage 4 denotes deep and extensive tissue damage involving muscle, tendon or bone. Hip and buttock sores represent 67% of all pressure sores.[1,2]

Rationality	Annual cost of treatment in the US exceeds $1 billion

Formula for calculation	Number of pressure ulcers / Number of cases X 1000

Patient population	Critically ill

Source of data	Hospital record

Type of parameter	Morbidity, Safety of patients

Bench mark	3 – 11%[1] 22.71 / 1000 cases[3]

References	Revis DR. Decubitus Ulcers. E Medicine October 25th, 2005. Pressure care for pressure ulcer and pain therapy. National Pressure Ulcer Advisory Panel (NPUAP). Available from: info@pressurecare.co.uk AHRQ national average. Sharphealth care 2007. Malcolm Baldrige National Quality Award application 2007. p. 34.

### 6.3 Operational or Process Parameters

#### 6.3.1 Length of Stay

**Table d32e1078:** 

Indicator	Length of Stay (LOS)
Description	Total hours and days patients managed in the unit with midnight bed occupancy are more accurate than the number of calendar days a patient spends in the ICU. Arithmetic mean overestimates LOS, as outliers both ways influence the mean LOS very adversely. **Median** of LOS can circumvent this problem. LOS is also influenced by factors such as availability of intermediary care, discharge practices, and mortality rates. Appropriateness of using LOS as outcome measure is therefore being reconsidered by Joint Commission on Accreditation of Healthcare Organizations (JCACHO). LOS properly stratified on the basis of diseases and conditions and properly analyzed could be a sensitive parameter throwing up deficiency in process and technique in ICU.

Rationality	ICU beds are limited in any hospital. Rationalized use for needy patients therefore is necessary. LOS is, therefore, used to assess quality of care and resource utilization.

Formula for calculation	Total occupied bed days / number of patients in a given time frame (weekly/monthly /yearly)

Patient population	All admitted patients in the unit

Source of data	ICU data

Type of parameter	Outcome measure

Bench mark	Average LOS in year 2001 Norfolk General Hospital[2] 4.36 days in general ICU; 2.43 days in vascular ICU

References	McMillan TR, Hyzy RC. Bringing quality improvement into the intensive care unit. Crit Care Med 2007;35:S59–65. Pronovost PJ. Accelerating Change Today (A.C.T.) for America's Health. Schoeni PQ, editor. © 2002 by the National Coalition on Health Care and the Institute for Healthcare Improvement. The Robert Wood Johnson Foundation supported report

#### 6.3.2 Compliance to Protocol

**Table d32e1164:** 

Indicator	Compliance to protocol
Description	Selected guidelines, protocols, treatment bundles in the unit to improve patient care, resource utilization, and reduce iatrogenic complications.

Rationality	Compliance to protocols, guidelines and treatment bundles are expected to improve patient care. Compliance to protocol could be absolute or partial (Seventy percent correct compliance had been reported by McMillan *et al*.[1])

Formula for calculation	Number of times followed/ number of times expected to follow × 100

Patient population	All ICU patients

Source of data	Audit report

Type of parameter	Process parameter

Bench mark	90%[2]

References	McMillan TR, Hyzy RC. Bringing quality improvement into the intensive care unit. Crit Care Med 2007;35:S59–65. Pronovost PJ. Accelerating Change Today (A.C.T.) for America's Health. Schoeni PQ, editor. © 2002 by the National Coalition on Health Care and the Institute for Healthcare Improvement. The Robert Wood Johnson Foundation supported report

#### 6.3.3 ICU Readmission Rate

**Table d32e1253:** 

Indicator	ICU readmission rate
Description	Readmission to the ICU within 24 hrs of transfer during a single hospital stay. This is an indicator of post ICU care.

Rationality	A zero readmission rate reflects a more *defensive* approach by the ICU team, which increases LOS in ICU causing risk of nosocomial infection, iatrogenic complications, and nonavailability of beds for the deserving patients. A higher mortality rate of 1.5 to 10 times that of controls, and higher length of stay at least twice that of control patients had been documented. A higher readmission rate indicates premature decision to shift out patients

Formula for calculation	(Number of readmitted patients/ Total patients managed in ICU) × 100

Patient population	All patients discharged from ICU in a time frame.(exclusion: death in CCU)

Source of data	Hospital record

Type of parameter	Process, Safety of patients

Bench mark	ICU readmission rates are around 5–6%[1] 4%[2]

References	1. McMillan TR, Hyzy RC. Bringing quality improvement into the intensive care unit Crit Care Med 2007;35:S59–65.
	2. Delgado MC, Pericas LC, Moreno JR, *et al*. Quality indicators in critically ill patients. SEMICYUC work groups. 1^st^ ed. May 2005. ISBN 609- 5974

### 6.4 Error and Patient Safety

Error is defined as “the failure of a planned action to be completed as intended, or the use of a wrong plan to achieve an aim”.[1] ^additional ref^ Culture of safety is important, considering the high number of preventable deaths (44000 to 98000/ annum medical error related deaths had been reported in USA).[2] ^additional ref^ Brochure released by society of Critical Care Medicine, USA in 2004 had quoted very high incidence of medication errors which caused more than 770,000 injuries and deaths per year.[3] ^additional ref^

Both patient safety and staff safety are important.

#### 6.4.1 Patients' Fall Rate

**Table d32e1371:** 

Indicator	Patients' Fall Rate		
Definition	An untoward event, which results in the patient coming to rest unintentionally on the ground or another lower surface.[1]		

Rationality	Fall could be accidental, anticipated physiological or unanticipated physiological. This is a safety issue for a patient in ICU. Accidental fall could lead to morbidity, prolonged stay and customer dissatisfaction.		

Formula for calculation[1]	fall rate = (no. of falls/no. of bed days) × 1000		

Patient population	All patients		

Source of data	ICU record		

Type of parameter	Safety and morbidity		

Bench mark[2,3]	8.46 falls per thousand bed days with an injury rate of 12.85% in 2000-01[2]		
	Norton Hospital USA, 2008 Norton Healthcare statistics per 1000 in patient days of the unit.[3]		
		Without injury	With injury
	ICU	2.10	0.22
	Medical surgical	2.23	0.74
	Medical	2.62	0.70
	Surgical	2.02	0.37

References	Barnett K. Reducing patient falls in an acute general hospital. In: Shaw T, Sanders K, editors. Foundation of Nursing Studies Dissemination Series. Vol. 1. 2002. Barnett K. Mid Yorkshire Hospitals NHS Trust. Reducing patients falls project. 2001-02 Norton hospital USA2008 Norton Healthcare. Available from: http://www.erlanger.org/quality/PatientSafety.asp		

#### 6.4.2 Medication Error

**Table d32e1516:** 

Indicator	Medication error
Description	Medication error could be due to wrong prescription, dosing or communication gap (verbal or written)

Rationality	Medication errors occur at a mean rate of 19% in hospitalized adults. The need for assessing ICU medication error frequency is highlighted by the finding that 78% of the serious medical errors that occurred in the ICU were attributed to medications. More than 235,000 medication errors were reported in 2003 in USA. At least 2% of these errors caused significant patient harm (eg. injury requiring treatment, prolonged hospital stay, and death.)[1]

Formula for calculation	Medication error rate = (no. of error /no. of bed days) × 1000

Patient population	All patients in ICU

Source of data	ICU record

Type of parameter	Patient safety

Bench marks[1,2]	Medication errors range from 1.2 to 947 per 1000 patient-days (median of 105.9 per 1000 patient-days) in adult ICUs and Median of 24.1 per 1000 patient days in neonatal/pediatric ICUs[1] Wrong dose: 105.9 errors per 1000 patient-days in ICU[2]

References	Kane-Gill S, Weber RJ. Principles and Practices of Medication Safety in the ICU. Crit Care Clin 2006;22:273–90. Herout PM, Erstad BL. Medication errors involving continuously infused medications in a surgical intensive care unit. Crit Care Med 2004;32:428–32.

#### 6.4.3 Adverse Events /Error Rate

**Table d32e1616:** 

Indicator	Adverse Events/Error R ate
Description	Common ICU errors are related to treatment, procedure, ordering or carrying out medication orders, reporting or communication, and failures to take precautions or follow protocols.

Rationality	Critically ill patients are at high risk for complications due to the severity of medical conditions, complexity of treatment, poly pharmacy and technology based interventions. Nearly all ICU patients suffer from potentially harmful events. Nearly half (45%) of the adverse events are preventable.

Formula for calculation	Adverse events/ error rate = (no. of error /no. of bed days) × 1000

Patient population	All ICU patients

Source of data	Medical record

Type of parameter	Safety (patient)

Bench mark[1]	The rates per 1000 patient-days for all adverse events, preventable adverse events, and serious errors were 80.5, 36.2, and 149.7, respectively. Among adverse events, 13% (16/120) were life-threatening or fatal; and among serious errors, 11% (24/223) were potentially life-threatening.

References	Rothschild JM, Landrigan CP, Cronin JW, Kaushal R, Lockley SW, Burdick E, *et al*. The Critical Care Safety Study: The incidence and nature of adverse events and serious medical errors in intensive care. Crit Care Med 2005;33:1694–700.

#### 6.4.4 Needle Stick Injury Rate

**Table d32e1699:** 

Indicator	Needle Stick Injury Rate
Description	A penetrating stab wound from a needle (or other sharp objects) that may or may not be associated with exposure to blood or other body fluids

Rationality	[1]Needle stick injuries can cause transmission of blood borne pathogens. Needle stick injury can occur due to faulty handling of needle, syringe with needle, suture needle, recapping of needle, and faulty disposal. Annual incidence ranges from 600,000 to 800,000 at global level. According to CDC estimate 385,000 needle stick injuries occur annually in the U.S. hospital settings. Approximately half of these go unreported.[2] Although this is a minor injury, transmission of disease is a concern. Blood filled hollow bore needles accounted for 63% of the needle stick injuries from June 1995 to July 1999. It is a preventable injury[3] therefore adequate training to health care provider is a must.

Formula for calculation	Incidence per 10,000 venipunctures

Patient population	All healthcare workers

Source of data	ICU record

Type of parameter	Safety (Healthcare worker)

Bench mark[2]	0.94 per 10,000 venipunctures USA national rate

References	Available from: http://www.medterms.com/script/main/art.asp?articlekey=25492. Rosenstock L. Statement for the record on needle stick injuries. Centers for disease control and prevention, Department of Health and Human Services Available from: http://www.hhs.gov/asl/testify/t000622a.html. [last accessed on 2000 Jun 2000]. American nurses' association's needle stick injury prevention guide. Washington, D.C: 2002-04. http://www.NursingWorld.org.1-800-274-4ANA.

#### 6.4.5. Reintubation Rate

**Table d32e1803:** 

Indicator	Reintubation Rate
Description	Reintubation within 48 hours of extubation

Rationality	Accidental extubation and subsequent reintubation can lead to prolonged stay, longer ventilation and higher nosocomial pneumonia and mortality

Formula for calculation	(Number reintubated/ Number extubated) × 100

Patient population	ICU patients with endotracheal tube had undergone planned extubation

Source of data	ICU record

Type of parameter	Morbidity, safety

Bench mark	12.2%[1], 12%[2]

References	McMillan TR, Hyzy RC. Bringing quality improvement into the intensive care unit. Crit Care Med 2007;35:S59–65. 2. Delgado MC, Pericas LC, Moreno JR, *et al*. Quality indicators in critically ill patients. SEMICYUC work groups. 1^st^ ed. May 2005. ISBN 609- 5974.

### 6.5 Infection Control

Nosocomial infection has both outcome and financial implications. Approximately 1.7 million infections, 99,000 deaths, and higher estimated annual expenditure of $4.5 billion had been reported by centers for disease control and prevention in 2007.[4] ^additional ref^ The three most commonly monitored variables are: a) ventilator associated pneumonia, b) blood stream infection and c) urinary tract infection rate were selected as quality indicators for this report. NNIS surveillance 2002 shows that out of the overall 13.04 overall infection rates / 1000 patient days in adult and children intensive care units, pneumonia, BSI and UTI rates were represented as 3.33, 2.71 and 3.38 respectively. SSI (0.95) and others (2.67) represented the rest. Percentage wise UTI, BSI, and Pneumonia incidence were 32, 14, 15 in the 2002 survey.[5] ^additional ref^

#### 6.5.1 Ventilator Associated Pneumonia (VAP)

VAP is an important cause of morbidity and mortality[6,7] ^additional ref^ but difficulties encountered in diagnosis of VAP makes bench marking a difficult proposition. Therefore its advantage as quality indicator is limited. Despite this hospitals in United States report ventilator-associated pneumonia rates as an indicator of quality of care and also for benchmarking. This is due to the collective demand of legislators, tax payers, and advocates of quality-of-care across.[8] ^additional ref^

Endotracheal aspirates with nonquantitative cultures had been advocated as the initial diagnostic strategy. Common clinical criteria (e.g. fever, leukocytosis, purulent secretions, new or changing radiographic infiltrate) have high sensitivity but suffer from relatively low specificity level. They are most useful for initial screening for VAP and for selecting patients for invasive procedures, such as BAL, that have sensitivities and specificities in the region of 80%. For ease of application clinical and radiological diagnostic criteria are given in this report.

**Table d32e1928:** 

Indicator	Ventilator Associated Pneumonia (VAP)	
Description	Ventilated patient developing new opacity and also fulfilling criteria of VAP	

Rationality	Ventilator associated pneumonia increases morbidity and mortality. It has cost implications as it increases days of ventilation. Reduction in the incidence rate is desirable in ventilated patients. Reported crude mortality rates in VAP exceed 50%, and the attributable cost of VAP approaches $20,000.[1]	

Diagnosis[2]	**Radiologic Signs**	
	≥ 2 serial chest radiographs with at least one of the following:	
	New or progressive *and* persistent infiltrate Consolidation Cavitation	
	**Clinical Signs (at least one of the following):**	
	Fever (temperature >38°C) with no other recognized cause Leukopenia (<4.0 × 10^9^ cells/L) or leukocytosis (>12.0 × 10^9^ cells/L) For adults ≥ 70 y of age, altered mental status with no other recognized cause	

	and ≥ 2 of the following:	
	New development of purulent sputum, change in character of sputum, increased respiratory secretions, or increased suctioning requirements New-onset or worsening cough, or dyspnoea, or tachypnoea Rales or bronchial breath sounds Worsening gas exchange (e.g., oxygen desaturation ratio [PaO2/FiO2] < 240, increased oxygen requirement, or increased ventilation demand)	

Formula for calculation	$\frac{\#\text{of patients with VAP}}{\#\text{of days mechanically ventilated with endotracheal tube}}\times 1000\text{days}$	

Patient population	All ventilated patients except neonatal intensive care patients	

Source of data	Hospital record of patient	

Type of parameter	Infection, outcome, safety	

Bench mark (1000 device days) NHSN[3]	Burn	10.7

	Coronary	2.5

	Surgical cardiothoracic	4.7

	Neurological	7.1

	Neurosurgical	6.5

	Surgical general	5.3

	Trauma	9.3

	Medical	2.5

	Medical/surgical, major, teaching	3.3

	Medical/surgical, all others	2.3

	Pediatric medical/surgical	2.1

References	Safdar N, Dezfulian C, Collard HR, Saint S. Clinical and economic consequences of ventilator-associated pneumonia: a systematic review. Crit Care Med 2005;33:2184-93. Horan T, Gaynes R. Surveillance of noscomial infections. In: Mayhall C, editor. Hospital Epidemiology and Infection Control. 3^rd^ ed. Philadelphia: Lippincott Williams and Wilkins; 2004. p. 1659-702. Edwards JR, Peterson KD, Andrus ML, Dudeck MA, *et al*. National Healthcare Safety Network (NHSN) Report, data summary for 2006 through 2007, issued November 2008.	

#### 6.5.2 Blood Stream Infection Due to Central Line

**Table d32e2214:** 

Indicator	Blood Stream Infection Due to Central Line	
Description	Blood stream infection rates = number of central line related BSI per 1000 central line-days	

Rationality	Bloodstream infection (BSI) had emerged as a major killer. The estimated death caused by BSI was 26,250 deaths/ year and it is ranked as the eighth leading cause of death in the United States.[1]	

Formula for calculation	$\frac{\#\text{Number of central line-associated BSI}}{\#\text{Number of central line-days}}\times 1000$	

Patient population	Adult	

Source of data	Hospital record	

Type of parameter	Infection, outcome, safety	

Bench mark NHSN[2]	Burn	5.6

	Coronary	2.1

	Surgical cardiothoracic	1.4

	Neurosurgical	2.5

	Surgical general	2.3

	Trauma	4.0

	Medical	2.4

	Medical/surgical, major, teaching	2.0

	Medical/surgical, all others	1.5

	Pediatric medical and surgical	2.9

	Pediatric medical	1.0

	Neurologic	1.2

References for Bench mark	Wenzel RP, Edmond MB. The impact of hospital-acquired blood stream infections. Emerg Infect Dis 2001;7:174-77. Edwards JR, Peterson KD, Andrus ML, Dudeck MA, *et al*. National Health care Safety Network (NHSN) Report, data summary for 2006 through 2007, issued November 2008.	

#### 6.5.3 Urinary Catheter Related Infection

**Table d32e2422:** 

Indicator	Urinary Catheter Related Infection	
Description	Incidence of UTI per 1000 catheterized days in patients catheterized in the unit but not infected on the day of catheterization	

Rationality	Prevalence wise, urinary tract infection is most common. It increases morbidity (if not mortality), cost and stay.	

Formula for calculation	$\frac{\#\text{Number of UTI}}{\#\text{Number of catheter days}}\times 1000$	

Patient population	All patients catheterized in the unit and were without infection on day one of catheterization	

Source of data	Data collected in the unit	

Type of parameter	Infection, safety, outcome	

Bench mark[1]	Burn	7.7

	Coronary	4.4

	Surgical cardiothoracic	3.2

	Neurosurgical	6.8

	Surgical	4.1

	Trauma	5.7

	Medical	4.1

	Medical/surgical, major, teaching	3.3

	Medical/surgical, all others	3.1

	Pediatric medical/surgical	5.0

References	Edwards JR, Peterson KD, Andrus ML, Dudeck MA, *et al*. National Healthcare Safety Network (NHSN) Report, data summary for 2006 through 2007, issued November 2008.	

### 6.6 Human Resource

Adequate and competent staff can ensure delivery of quality oriented service. Therefore, adequacy of human resource and its development are important issues. The unit should pay attention to monitor attrition rate. Leader of the team should interact with internal, external agencies, ICU staff to ensure delivery of predecided standard of care. Positive culture to encourage innovation, autonomy, empowerment, safety, ethical standard, staff satisfaction, should also be developed to achieve goal of the unit and the organization. Overall employee satisfaction is advocated in this report.

#### 6.6.1 Overall Employee Satisfaction

**Table d32e2610:** 

Indicator	Overall Employee Satisfaction
Description	Satisfaction level of the staff working in the hospital/unit

Rationality	Satisfied work force gives better output. Retention rate remains high

Formula for calculation	On a 1 to 5 point scale where 1 represents lowest satisfaction and 5 indicates highest possible satisfaction

Population	Staff working in the unit

Source of data	Employee satisfaction survey

Type of parameter	Human resource

Bench mark	4 score best in class

References	Sharp health care, 2007 Malcolm Baldrige National Quality Award application 2007,

### 6.7 Customer Focus

Perception of patients and their relatives about the care received is an important determinant for forming public opinion. If care perceived is not good, it causes customer (patient, relatives) dissatisfaction. Patient's and family's satisfaction level should never be ignored and regular attempts to assess the gap between actual level of care (based on the survey by healthcare provider and other quality parameters discussed above) and perceived level of care(customer dependent), should be made. Patient satisfaction is included for this report. In units where most of the patients, due to their physical condition, are not in a position to give feedback, relative's opinion can be taken.

#### 6.7.1 Patient Satisfaction (Customer Satisfaction)

**Table d32e2689:** 

Indicator	Patient Satisfaction
Description	Patient satisfaction is a perceived parameter by the patient.

Rationality	Reflects performance of the hospital as perceived by patients (customer) Satisfaction of the customer is directly related to financial return to the hospital and also reveals institutions credibility in the population it functions. It also gives opportunity for improvement.

Formula for calculation	No formula is available to us. Survey can be conducted by external agency to eliminate bias or on regular basis feedback forms can be collected for analysis. Feedback forms should address areas such as: admission/registration process, facilities, food, interactions with nurses and physicians, discharge process, personal issues, overall assessment of the care and other services Feedback form with 10 point scale can be used where 10 is for the best possible service. Patients give a rating for all the questions. Overall mean (average) score for each service is calculated from the rating given by each patient.

Patient population	All patients admitted in ICU and capable of giving feed back

Source of data	Feedback forms

Type of parameter	Customer focus: Perceived quality of service

Bench mark	Satisfaction level to physician's communication (always well communicated?) = 80%, nurses' communication (always well communicated?) = 74%, cleanliness (always clean) = 70%, noise (quiet at night?)= 56%, responsiveness (always responded?) = 63%, pain management (always well controlled?)= 68%, communication about medicine (always explained) = 59%, discharge information =80%, will you recommend the hospital? = 68%, is it the best hospital? = 64%

National average USA 2008	Note: Methodology of collecting data influences the parameter and, therefore, uniformity is essential.

References	Available from: http://www.erlanger.org/quality/PatientSatisfaction.asp

## 7. Dashboard (Annexure 14.7)

Based on the selected parameters, a dashboard has been prepared for systematic data entry of the parameters. Participating centers are expected to use the Dashboards and send the same to the central body for review, analysis, and to collate available data for preparing a national data base. Following acceptance of this Dashboard, formula for automatic calculation of performance parameter will be incorporated.

## 8. Limitations

Very common parameters have been selected in this report. Stress has been given on mortality, morbidity, infection and safety of patients. Acceptability and utility of these parameters in the Indian scenario will have to be assessed over a period of time.Diagnosis of VAP is controversial. Clinical and radiological diagnostic criteria are given in this report for ease of application in the Indian scenario.Compliance to protocols had not been given more importance because during the initial stage; monitoring will be difficult in most of the units and therefore generation of corrupt data is possible. Similarly, only overall satisfaction of employees has been suggested in this report even though satisfaction level can be judged by various means.Certain institutions may have reservations in sharing their data base, while due to lack of logistic support many institutions might find difficulty in generating regular and meaningful data.Considering wide variability of practices and resources in Indian ICUs, the initial data base might not represent actual level of care in quality oriented units in India.Till the national data base starts generating data specific to specialized units, comparison for such units will be difficult.All bench marks included in this report do not represent national bench marks. Whenever national bench marks could not be found, bench marks have been taken from the figures given in reputed journals but these could be different from the national averages.All participating institutions might not be comfortable in monitoring all the suggested parameters.NICU related bench marks have not been mentioned in the report.

## 9. Future Course of Action (as suggested by this task force)

This report is only the beginning of the broad based objective of quality orientation in the Indian scenario. Future direction, therefore, is necessary to achieve its final objective.

### 9.1 Generation of National Data Base

Authenticity of data due to a variable system of data collection (manual vs. electronic), variability of practice, infrastructure, support etc will be the biggest challenge in forming a national data base. Institutions, therefore, will have to be selected based on prefixed guidelines for contribution to the national data base. Compliance to national level guidelines, surveillance system, reporting frequency set by ISCCM and commitment to provide correct data should be part and parcel of such guidelines. Steps will be necessary to ensure data collection, collation, stratification and analysis of data, to make it meaningful for the end user. Responsibility will have to be given to a central body / institution with adequate support to accomplish this job on regular basis.

### 9.2 Data Base for Specialized Units

This is a future consideration so that national data base is available for units looking after a specific subset of patients.

### 9.3 Inclusion and Exclusion of Parameters

Many new parameters will have to be included to address the need of the ICUs managing specific subset of patients and accordingly national bench marks will have to be decided. All the institutions might not be willing to compare their results against all the parameters selected at a national level. Liberty should be given to the institutions to select a few optional parameters while monitoring mandatory parameters. Decision will have to be taken to identify mandatory parameters. Utility of certain parameters over a period of time might have to be questioned and decision to take them off the dash board will have to be taken from time to time. This happens when the unit appreciates that the parameters are not helping any more in bringing a positive change.

### 9.4 Evaluation of Performance level

Institutions can match their performance against the national data base. While granting accreditation to institutions for the Indian Diploma for Critical Care (IDCC)/fellowship in critical care and inclusion as participating institutions for the national data base, performance level of the institution should be taken into consideration. A national data base subsequently may be compared with international bench marks.

### 9.5 Research, Qualitative Improvement

A national data base can be used for improvement cycles (“Plan – Do- Check - Act” i.e. PDCA) to bring qualitative improvement in the unit and even at the national level.

### 9.6 Public Reporting

Public reporting should be the ultimate objective of the whole exercise so that patients and their relatives can take a conscious decision while selecting an institution for its offered services and performance levels. However, to prevent misuse of the national data base and inappropriate projection for boosting the image of the institution or financial gain for the institution; **right to use the data base should be restricted**. Public reporting should be allowed only with prior approval of the “ISCCM quality parameters body,” which could be formed and authorized to grant permission.

### 9.7 Audit System

Periodic auditing of institutions interested in the post doctoral course contributing data for the national data base and public reporting of their performance against the national data base, should be done to maintain uniformity of standards set by ISCCM. An audit team should be formed by the national body to address this issue. Methodology for auditing, scoring systems and a cut-off limit should be set for this purpose.

### 9.8 ISCCM Quality Parameters Body

Formation of a team will be essential to address various aspects related to maintaining, updating data, formation of an audit team, audit schedule, auditing system, training, amendments in national dashboard, inclusion, exclusion and modification of parameters and to address any dispute related to quality parameters.

### 9.9 Benchmarks

Periodic amendment of bench marks given in this report will be necessary with the availability of newer bench marks from developed countries and a national data base.

## 10. Recommendations

Following approval of this report, pilot implementation of advocated dashboard in selected few institutions is recommended with monthly updating of the dashboard.Based on the experience of the participating institution and central body while developing the national data base, further amendments can be done.Future steps suggested above can be considered in a phased manner subsequently.

## 11 List of Symbols

**Table d32e2878:** 

Symbols	Definitions
**Agencies:**	
AHRQ	Agency for Healthcare Research and Quality
JCAHO	Joint Commission on Accreditation of Healthcare Organizations
NNIS	National nosocomial infection surveillance system
CDC	Center for disease control and prevention
**Terminologies:**	
ISCCM	Indian Society of Critical Care Medicine
SMR	Standardized mortality rate
APACHE	Acute physiology and chronic health evaluation
MPM	Mortality prediction model
BUN	Blood urea nitrogen
GFR	Glomerular filtration rate
ARF	Acute renal failure
LOS	Length of stay
VAP	Ventilator associated pneumonia
BSI	Blood stream infection
UTI	Urinary tract infection (catheter induced)
**Units:**	
ICU	Intensive care unit
BCU	Burn care unit
CCU	Coronary care unit
SCU	Surgical cardiothoracic unit
MICU	Medical intensive care unit
M-S ICU	Medical/Surgical, major, teaching intensive care unit
PICU	Pediatric intensive care unit
Neuro (Med) ICU	Neuro(medical) intensive care unit
Neuro (surg) ICU	Neuro (surgical) intensive care unit
SICU	Surgical intensive care unit
TICU	Trauma intensive care unit

## 12. Acknowledgment

The task force members compliment the national executive body for considering lack of quality indicators and a national data base as an important issue. It was an honor and privilege for the members to be a part of this exercise, which is the first of its kind at a national level and particularly in the field of critical care. This is a humble beginning for a mammoth task waiting to be completed. As chairman, I express my deepest gratitude to each member of the task force for his valuable contribution in finalization of this report.

## 13. Additional References

Kohn L, *et al*. Washington, DC: National Academy Pr.; 2000.To Err is Human: Building a Safer Health System 1999 by Institute of Medicine (IOM).Improving your ICU, Tips for better care. Society of Critical Care Medicine USA in 2004 Available from: info@sccm.orgThe Center for Disease Control and Prevention estimates of health care associated infections. Available from: http://www.cdc.gov/ncidod/dhqp/hai.html. [last accessed on 2007 Oct 15].Klevens RM, Edwards JR, Richards CL, *et al*. Estimating Health Care-Associated Infections and Deaths in U.S. Hospitals, 2002. Public Health Reports / March–April 2007 / Volume 122.Heyland D, Cook, Hamilton ON, Dodek PD, *et al*. The Canadian Critical Care Trials Group, A Randomized Trial of Diagnostic Techniques for Ventilator-Associated Pneumonia. N Engl J Med 2006;355:2619–30.Muscedere J, Dodek P, Keenan S, Fowler R, Cook D, Heyland D, *et al*. Comprehensive evidence-based clinical practice guidelines for ventilator-associated pneumonia: diagnosis and treatment. J Crit Care	2008;23:138–47.Klompas M, Platt R. Ventilator-associated pneumonia—the wrong quality measure for benchmarking. Ann Intern Med 2007;147:803–5.

## 14. Annexure

14.1. Quality indicators in, critical care: An overview. B Ray and D P Samaddar

14.2. Patient safety. S K Todi

14.3. Personnel development. Suresh Ramasubban

14.4. Quality of processes. George John, and N Ramakrishnan

14.5. Outcome parameters. George John, Dr N Ramakrishnan

14.6. Infection control Dr D P Samaddar

### 14.1 Quality Indicators in Critical Care: An Overview

**B Ray, D P Samaddar**

Quality of care in medical practice in general and critical care in particular is the responsibility of the care provider. Clinicians involved in providing the care are, therefore, morally and ethically bound to enhance quality. Level of care varies among ICUs and within ICU. Even small adjustments can significantly improve quality of care and patient outcome.[1] Care before and after improvement initiatives can be quantified provided attributes to measure the care are predefined. Quantification of ICU performances, is not an easy task because it depends on multiple variables involving medical knowledge, ethics, economics, systems, engineering, sociology, and philosophy.[2] Regular monitoring of parameters is a labor intensive process. Therefore, selection of quality indicators and prioritization should be done to ensure maximum impact with minimum data collection.

**Objective:** Monitoring of quality indicators is done to identify level of care provided on a time scale. Trend analyses of such data helps in quantifying the standard of care offered in the same setup and compare the same with selected bench marks. Improvement initiatives are subsequently taken, to bridge the gap between the levels offered and bench mark levels, as per need.

The issue of quality indicators in ICU will be discussed under two broad headings in this article

#### A: Conceptual Basis

#### B. Selection and Implementation of Parameters

**A: Conceptual Basis of Quality Indicators:** Quantification of parameters must have relevance to patients, hospital and the society. Before selecting indicators it is therefore important to understand the conceptual basis of quality in critical care.

#### Outcome Parameter

Success rate of the unit is of paramount importance. The basic reason of providing healthcare is to improve outcome. Survival / mortality rate of the unit makes significant impact on the relatives of the patients, hospital authority, and the general mass in that locality. Credibility of the unit is judged to a great extent by its mortality statistics.[3] However, crude mortality is not a sensitive parameter. If moribund and terminally ill patients mostly get admitted in the unit because intensive care is being used as the dumping ground then mortality naturally will be higher. Contrary to this, higher success rate will be observed if the unit manages patients who are mostly not serious enough to deserve intensive care admission. Measuring the crude mortality rate therefore can be misleading if it does not address changing patient profile.[4] For better assessment, mortality should at least be correlated with severity status of the patients and disease state (case mix).[2] Hence some scoring system should be in place to stratify severity status of the admitted patients and link it with the outcome.

**Morbidity Indicators:** Unanticipated developments or iatrogenic complications (examples given in Table 1) indicate cost of poor quality (COPQ). Morbidities have important bearing on the ultimate outcome, resource utilization, length of stay (LOS) and subsequent quality of life patient enjoys. Monitoring of morbidity and steps taken to reduce the incidence help in achieving the primary goal of better outcome. LOS and better resource utilization are secondary objectives which automatically get addressed at least to some extent with such approach.

**Table 1 T0003:** Suggested Measures of ICU Performance

	Indicators	Parameters
1	Mortality	ICU: Crude, Severity adjusted, Disease based Hospital mortality
2	Morbidity	**Incidence of:**
		Accidental extubation, Reintubation in planned extubated patients, Pneumothorax,
		Unanticipated cardiac arrest, hypotension, renal failure
		%Nosocomial infections (VAP, BSI, UTI)
		%of patient with VRE, MRSA.
		% of patients with GI bleed
		Pressure sore, dental trauma, nerve and vascular injury.
		ICU readmission within 24 hours
3	Cost Effectivity	**Patient/ICU day cost to the institution**
		Actual expenditure on Man power cost, capital equipment cost Equipment maintenance, consumables, Diagnostics, house keeping, electricity etc.
		Overall expenditure in ICU
		Expense (post ICU) in hospital
		Expense (post hospital) after discharge
		Long term survival and quality of life
		Per survivor cost in ICU/ hospital / post discharge
4	Safety of Patient	Error reporting: incidence of different errors
		Complication rates related to care
		**Number of complications/ patient**
		Incidence of mishaps during transportation
		% compliance to waste disposal
		% compliance to hand hygiene protocol
		Blood component therapy
		Frequency of noncompliance to protocol.
		Antibiotic free stay (days) in ICU
		Antibiotic resistance and Drug resistant microbial pattern
		Broad spectrum antibiotic use/1000 patient days
		De escalation in % of patients receiving antimicrobials
5	Safety of ICU personnel	Number of needle stick injuries
		Number injured while working
6	Man power	Per Person training (in hours or days)/Yr Appraisal of targets given.
		Staff satisfaction and turnover rate
7	Resource utilization Infrastructure	**ICU:**
		Number of patient managed, %bed occupancy, **Av. LOS**, total occupied bed days, % ICU patient ideally should be shifted but remaining in ICU, number of readmissions, fraction of patients for whom ICU care is expected to be futile, number of X- rays done / 1000 patient days. **Average ventilatory days**
	Equipment	Utilization in days or hours/ month
		Downtime in days or hours/ month
		ROI (Return on Investment) of individual equipment
8	Customer external	**% satisfaction level of patient**/relatives
		Number of negative and positive feed backs
		Number of complains/ suggestions and number addressed.
	Internal Customer	Satisfaction of others in the hospital with the care and services supplied by the ICU.
9	Administrative	Revenue generation

**Readmission and Length of Stay:** Judicious transfer of patients to ward is important to prevent overstay. On the contrary, overzealous and injudicious shifting can lead to readmission and mortality. Mortality of 1.5 to ten times and twice the length of stay (LOS) in readmitted patients, (compared to control patients), has been documented in the literature. Premature transfer can reduce ICU stay and expenditure[5] but at the cost of worse outcome.[6–8] LOS therefore should also be correlated to ICU readmission within 24 hours of transfer during a single hospital stay. Reported ICU readmission rates are around 5–6%.[9,10] Readmission rate of a given setup can be compared with such bench mark data. Reduction in ICU readmission rate can be taken as improvement initiative to reduce crude mortality. While doing so, a root cause analysis should be done so that vital few causes are addressed primarily to get maximum benefit. Caution is necessary while drawing conclusion from readmission data as many ICU readmissions are due to poor post shifting care in the ward therefore linking ICU readmission to injudicious decision making and quality of care in ICU would be illogical under such circumstances.[2] Target taken to reduce such readmissions will make ICU team more defensive leading to prolonged and unnecessary ICU stay. Higher risk of nosocomial infection and iatrogenic complications and creating a strain on hospital resources will be the end result of such defensive approach.[4] LOS of ICU is not a very sensitive parameter unless it is linked with ultimate outcome. Short-term outcomes like LOS should therefore be correlated to long term outcomes at least in the hospital and preferably on a more long term basis such as survival adjusted for the quality of life (quality-adjusted life years).[11]

**Cost Effectiveness and Revenue Generation:** Resources are not unlimited. Higher expenditure in ICUs is a global concern. One day in an ICU costs $2,000 to $3,000, which is six-fold higher than those for non-ICU care.[5,12] This is more important if patient, generally entertained in a given unit belonged to poor socio economic status, not covered by medical insurance and also not supported financially by state for free medical care. This kind of situation is more often a rule than exception in India. Considering this background, ICU expenditure/ patient/day is an important parameter. Attempts should be made to minimize it by taking local factors into account while practicing evidence based medicine and international protocols. Cost conscious units can maintain the same quality or offer a better quality with lesser and judicious utilization of resources. There is no proportionate relationship between the cost and quality. USA, despite being the most expensive medical care system, is not the leading nation in quality of care. Cost effectiveness is expected to be the natural fallout of efficient care. The benefit so accrued can either be shared with the patients/ relatives by maintaining same quality at reduced charges or enhancing offered service level without reduction in charges.

Economic viability of the unit is judged by the income generated after deduction of all expenses. Higher management always measures the success in terms of revenue loss or gain. Analysis of the expenses to identify expenditure on vital few and trivial many should be done. Measures should be directed at vital few items to get maximum return. While doing this exercise, judicious cost control should be done. Prevention of wasteful expenditure can significantly reduce running cost of the unit.

**Resource Utilization:** Because ICU care is expensive, resource utilization should be optimum. Assessment of resource utilization should be reflected in selection of quality indicators.[2] Optimum utilization of beds is essential to make the unit economically viable. Number of patients managed, percentage occupancy, average length of stay (LOS) and occupied bed days (LOS of each patient added in a predefined duration) etc. help in quantifying resource utilization and justifying the need of future expansion. Deserving patients denied ICU care due to paucity of bed or equipment, percentage of patients remaining in ICU who could have been managed elsewhere and patients getting ICU care where intensive care is expected to be futile should be taken into consideration while assessing judicious utilization of resources.[13–15] Adherence to written or published ICU admission and discharge standards can be used to measure the quality of ICU bed utilization, but such standards have not been subjected to the scientific validation and therefore are not endorsed for this purpose.[2] Proprietary systems such as APACHE III can be used to match unit data with the predicted ICU length of stay, days spent receiving mechanical ventilation, and the likelihood of receiving active intervention. This approach is limited by the fact that APACHE III has been validated only for the length of stay.[16] Despite the limitations in addressing this issue, local protocol should be developed based on the scientific background and local factors.

Equipment utilization is an equally important dimension of resource utilization to justify future procurement. Downtime in hours, revenue loss due to equipment remaining down, expenditure on overall maintenance of equipment and equipment wise revenue generation (return on investment or ROI) indicate the efficiency of maintenance support and skilled utilization of equipment by the ICU team. Check list of all equipment should be updated in the unit on a daily basis to monitor equipment utilization and downtime.

**Errors and Patient Safety:** Focus should be both on safety of patients and care providers. The 2005 Critical Care Safety Study, published in the August 2005 issue of *Critical Care Medicine,* reported that adverse events in ICUs occur at a rate of 81 per 1,000 patient-days and that serious errors occur at a rate of 150 per 1,000 patient-days, supporting the findings of an earlier study indicating that nearly all ICU patients suffer potentially harmful events. According to another study conducted in an Israeli ICU, errors were observed to occur in 1% of all the activities performed each day and incidence was higher with physicians than nurses.[5,12] Nearly half (45%) of the adverse events were deemed preventable in the Critical Care Safety Study.[17] Medical errors and hospital-acquired complications often lead to disability, large costs and mortality. 27,000 to 98,000 preventable deaths/ year had been reported in USA due to medical errors which is a matter of great concern.^[18–22]^ The situation is not expected to be better in other countries. Common ICU errors are treatment and procedure related. Medication errors result in more than 770,000 injuries and deaths each year at a cost of up to $5.6 million per hospital, depending on size in USA.^[23]^ Communication failures, while ordering prescription or carrying out medication orders and compliance to protocols, are often the important causes of errors.^[24–26]^ A more disturbing fact is the denial by physicians and nurses that the error was committed by them. In one study, one-third of ICU nurses and physicians denied having erred in the ICU, whereas at the same time they said that many errors are neither acknowledged nor discussed.[5,12]

Errors could be due to various reasons. Shortage of man power, deficiency of trained manpower, injudicious work pressure, inadequate infrastructural and equipment support, lack of protocol, and personal issues are the few important causes of errors. These factors should be addressed before blaming a person. Complacent attitude and lack of commitment could also be responsible for certain errors though it is infrequently observed in a sensible unit.^[23, 27]^ Most of the errors are not caused by individual inadequacies but are a product of defects in the system of care.[4] Therefore, before conducting an error surveillance, ambiguity of practice in offering various services should be eliminated. Care providers must know what is expected from them. Guidelines, protocols, systems and processes developed locally with reference to national/international guidelines and recommendations should be in place.^[23]^ Protocols should be in written form and adequate training should be given to the people who are expected to follow the protocol.^[28]^ Development of local guidelines/processes etc. should be done in consultation with the stake holders to break the resistance and to create a sense of ownership. This exercise should be done in piecemeal and training should be imparted as the systems and processes are being developed and implemented. Noncompliance to monitoring and record keeping should be done regularly to find out the magnitude of problem and area of concern.^[29]^ Writing protocols is relatively easy but implementation of the same and to conduct compliance monitoring are difficult to accomplish. Non-adherence to established standards of care have been related to poor outcome.[4] Only 50 to 70% of Americans receive the care that is recommended for their condition^[30]^ and 20 to 30% receive inappropriate medical interventions.^[18, 31–34]^ Parameters should also be selected to ensure that care providers are not exposed to undue risk. Audit can also be done at prefixed intervals by internal and external agencies to find the safety standard of the unit. Corrective measures can be taken accordingly. In authors' view, error documentation and analysis is expected to pay rich dividend in quality oriented and matured unit where acceptance of deficiency is not considered as a failure rather viewed as an improvement opportunity. A more practical approach for beginners would be to select mortality, morbidity and resource utilization parameters. Introspection drive for error documentation and analysis can be given priority when the unit is ripe enough to accept the deficiency without being defensive about it.

**People:** Efficient, motivated and trained man power is the backbone of any critical care unit. Training is must for maintaining and further up gradation of skill of the ICU personnel. Imparting training based on identified need is essential for any sensible unit. Days or hours of training should be monitored as a parameter.^[29]^ Effectiveness of training in the form of reduction of repetitive errors is, however, the end product of good training.

Although certification for critical care nurses is not mandatory, certification comforts patients and employers that a nurse is qualified and had gone through rigorous training requirements to achieve the additional credential.^[23]^ The same is true for the doctors involved in the unit. Efficiency of work force should also be monitored based on the targets given to them in relation to certain key result areas.

The satisfaction level of staff is very important. Higher turnover due to dissatisfaction causes wastage of time and money on staff training. Quality of care goes down due to higher turnover. Replacement of trained and motivated manpower is not good for the unit. Satisfaction level and staff turnover should therefore be taken as performance parameter of the ICU.[2] Many survey tools are available to assess this aspect.

**Customer Focus:** Care provided should be perceived and appreciated by the patients and relatives. Concern and empathy should be exhibited by the natural action of the care provider. ICU patients or their surrogates are often dissatisfied with the amount, nature, and clarity of communications by care givers. These contacts, which are often delayed and too brief, lead to confusion, conflict, and uncertainty about the goals of therapy.[2] Communication protocol and complain capturing and handling system prevents confusion and conflict. Patients and their relatives should be encouraged to give suggestions and to express their feelings. Number of complains/suggestions lodged and addressed could be taken as parameters. The mere distribution of feed back form, though, is easier and often does not serve the purpose if the educational background of feedback givers does not match the expectations of the surveillance team. Instead of routine ritual of passive surveillance, effort should be made to explain and assist the relatives of patients or patients before giving them feed back forms. They should also appreciate the need and importance of surveillance otherwise they might ignore such request. They should also be encouraged to give feed back without hesitation and fear. Such active surveillance is expected to be a better alternative and helps in identifying actual difficulties and expectations of the target population. Uninhibited feed back is possible if care providers are not part of such surveillance. Trained third party involvement for conducting the survey and analysis is a better but a costlier alternative. Care providers can help in designing the feedback format based on the past feed backs and area needing more attention. Efficient customer feed back system also helps in identifying expectations of the community. Quality indicators should be selected keeping these concepts in mind. Frequency, method of surveillance and analysis should be predefined. ICU management should do compliance monitoring and keep the necessary documents for review. Corrective action taken should get reflected in the subsequent satisfaction survey provided methodology remains the same.

**Variation in standard of care:** Variation in care is mostly due to geographical location, type of hospital, and physicians' preference. These variations can be tackled to a great extent by protocols developed based on international guidelines and evidence based medical approach.

Resistance offered by individual clinician or group of clinicians could be the road blocks while implementing the protocols and systems. Protocol-based approach might be viewed or projected as restriction in the authority and autonomy of individual clinician but keeping objective of evidence based medicine in view such variability should be curbed. Supportive administration can help in overcoming such resistance. While developing local protocols, individuals' or groups' opinion should be honored as much as possible. Once protocol is developed, compliance of these is expected from them. Monitoring of compliance and need based action is the responsibility of ICU management with the help of hospital authority.

Variation in care due to financial status, and insurance coverage could be difficult to address. In one study, 200 to 400% variation was noted in the use of pulmonary artery catheter due to ICU organization and staffing pattern, 38% due to racial variation and 33% was in relation to patients' insurance status.^[34]^

## B: Selection and Action Plan for Implementation of Indicators

**Selection:** It is practically not possible to select all the parameters discussed above. Therefore, while selecting the performance measures certain basic principles should be kept in mind.[2]

Evaluate varieties of parameters that cover the dimensions of ICU performance.Select performance measures that are primarily relevant or that have a proven relationship with the primary objective of the unit.

While selecting parameters focus should be on the ultimate outcome of the patient. Therefore, selection of mortality parameters is mandatory. High impact morbidity parameters should also be taken into account to reduce mortality. Resource utilization and financial results are essential from administration point of view. Similarly satisfaction level with the care and communication is of paramount importance to judge the customers' perception. Critical care team members generally need not test the validity, reliability, and responsiveness of every quality indicator they choose, but they should ascertain that attributes of the indicators have linkage with the objective of the unit.^[35]^

Following this, other parameters which have a bearing on morbidity and mortality, (such as error reporting and analysis, safety, compliance monitoring, training etc.) should be included based on the specific requirement of the unit. Willingness and courage to accept criticizm and desire to enhance quality through self introspection are prerequisites for error reporting and analysis. Mutual understanding between doctors, nurses, other staff and ICU directors is also important for embarking on error reporting exercise. During error reporting, it is imperative to understand that despite potential relationship of errors/adverse events with morbidity, mortality and cost^[19]^ all events do not lead to clinically relevant consequences.[11] Adverse drug events (ADEs) should always be monitored^[23]^ due to its direct linkage with morbidity. Underreporting and surveillance problems make the data collecting system porous. It has been claimed that incident reports, or chart reviews are inefficient, inaccurate, and debatable means of data collection.[4] Similarly cultivation of safety consciousness is also essential before safety practices are introduced and parameters are selected to monitor the safety standards.^[24]^ Safety self assessment and personal safety plan helps in paving the way for bringing a safety consciousness in the unit.

## Action Plan

**1. Target Setting and Benchmarking:** Current level of performance and bench mark data help in deciding the future targets. For example, if reintubation rate is considered the perceived problem and needs attention, the gap between the current level of reintubation in the unit and bench mark should be identified. Literature background of bench mark and method adopted for collecting the data should also be noted for future reference. Reported reintubation rate in patients receiving mechanical ventilation is 12.2% within 48 hours of extubation based on the published data of large international survey conducted by Esteban *et al.* in 2002.^[36]^ This target can be used as a benchmark, provided unit is planning to collect similar data for comparison. An overstretched but realistic target should be selected with appropriate action plan to achieve the target. However, such an approach cannot be used always because database is scarce and incomplete; therefore comparing each parameter might not be possible.^[37]^ Comparison with the unit's own data can be done in such situations. Moreover, influence of nonphysiologic parameters such as socioeconomic factors should not be ignored while linking the monitored parameter with the main objective i.e mortality, morbidity outcomes.[2] Due to these reasons, direct comparison with the bench mark data from a different socio economic background might not be always logical.

Reducing the incidence might not be the desired goal always. For example reducing the readmission and reintubation to zero level would be ideal but would be associated with unnecessary stay and prolonged ventilation respectively.[4] Sometimes, availability of an appropriate bench mark could also be difficult. If 41% reintubation rate in unplanned extubation in the above mentioned survey is compared with 12.2% reintubation in planned extubation, then it becomes evident that deciding optimal time of extubation and acceptable rate of reintubation is not an easy task.^[38]^ Similarly, it is not plausible that error levels will be zero; the goal should be reduced error rate to an acceptable level or below what it was previously present in the setup. Presence of error does not always prove that the overall performance is poor; therefore target setting and interpretation of result should be done with care.[2] The Joint Commission on the Accreditation of Healthcare Organizations (JCAHO) has developed eight ICU Core Measures. Ready made tools are also available that can assist in measuring individual unit's performance. [Available at: JCAHO Project Impact CCM, Inc.]

**2. Data Collection:** Hospital Management System (HMS) should be robust enough to generate data and analyze the same based on the fed information to minimize man power utilization and errors.[4] Information collected by the computerized system is superior to that collected by humans, especially if the system is specifically programmed to acquire the desired information. ICU team should remain involved if a tailor-made soft ware is being used. Specific need should be identified and introduced by ICU professionals while the software is being developed.

Manual data collection is possible but computerized physician order entry (CPOE) system automatically detects errors in unbiased manner and thus improves quality care by reducing costs and errors.[2, ^23^] In the absence of this, data collection and incidence reporting by individuals is the only viable alternative. Predefined criteria for data collection should be established with least dependency on human judgment. It is always better if ICU personnel collect data rather than relying on the health-care workers.[2] Ownership and accountability should be fixed for data collection, monitoring and maintenance of score board.

**3. Trend Analysis:** Score Card should be prepared to accommodate vital parameters based on the monitored parameters. Score Board should depict overall performance of the ICU. This helps in the systematic collection of data, monitoring of important parameters at a glance and also conduct trend analysis. While selecting the parameter, whenever possible correlate the desired parameter (numerator) with another parameter (denominator) to make it more meaningful. For example number of adverse events (numerator) can be expressed as the rate of events by dividing the absolute number with a denominator like aggregate number of at-risk patients, patient-days etc.[2]

Irrespective of the data type, care must be taken to collect a sample size that is large enough to allow reliable statistical comparisons.[2] Suppose monthly tracking shows that a particular parameter fluctuates between 0 and 6%, then while doing the trend analysis over a period of time a difference in the parameter within the acceptable limit should not be considered as deterioration in service. Thus, it is important not to over-interpret short-term changes in performance measurements while evaluating the same.[2] Frequency of data analysis is therefore important. Short term analysis can show wide variation in the parameters.

Standardization and accuracy of data collection is also important for subsequent analysis and comparison. For example measuring the number of calendar days a patient spends in the ICU is likely to overestimate LOS. Accuracy will be better if exact number of hours occupied or the number of days with midnight bed occupancy is taken into account for LOS calculation.^[39]^ A proper statistical analysis is also important for avoiding misrepresentation of data. If the arithmetic mean is used to calculate LOS in the ICU, it will often misrepresent the population because LOS data are skewed by atypical stays of few patients. Reporting the median, mode, or geometric mean will more accurately reflect the central tendency of the data.^[40]^ The standard deviation and range will also be informative while interpreting LOS data and instituting improvement initiatives.

**4. Continuous Improvement:** Data collection alone, by any means, is an insufficient guarantor of the delivery of quality medical care because it merely facilitates and does not ensure a predefined care unless process modification and corrective actions are taken for continuous improvement.[4] **A**nalyzed data indicates the direction the care provider should take to bring a positive change. PDSA cycle (Plan, Do, Study, and Act) should always be followed to bring a qualitative change in the performance. This cycle is repeated after achieving the desired goal and while setting new goals.^[41]^ Common tools used while following the PDSA cycle are: brain storming, cause and effect diagram, prioritization of ‘vital few’ causes, corrective action, and monitoring of impact. Process improvement is needed even for data collection to prevent it from becoming burdensome.[4]

**5. Team Building:** Contribution of ICU team and involvement of each member is vital for qualitative and sustained change in the unit. It is also important to appreciate that a close working group of dedicated healthcare providers can be as, if not more, important than the written protocol.[4] ICUs offering a “closed” model of care (ICU care and admission and discharge decisions made exclusively by intensivists or in consultation with intensivists), have shown better outcome parameters and shortened LOS.^[42]^ An association of leaders of industry from the Business Roundtable, **the Leapfrog Group**, has advocated for the widespread implementation of the intensivist model of care in the ICU.^[43]^ The same recommendation had subsequently been given by the **National Quality Forum**.[4]

**Conclusion:** Quality indicators act as the yard stick to measure the level of care offered in a unit over a period of time. Variation in care in the unit and among different units with similar case mix can only be done if indicators are compared on regular basis. Quality of care in ICU depends on the complex interaction between patient, machine and care providers. Process driven and protocol based management should eliminate ambiguity and ensure better outcome. Such approach is not possible unless care provided is quantified and gap between current level and desired level is assessed followed by improvement initiatives taken to bridge the gap. Selection of indicators and monitoring the same should, therefore, be considered the most vital and challenging task to bring continuous improvement in the performance level of the unit.

## References

Parker MM. A letter from the president. SCCM President, 2004 SCCM President, 2004.Garland A. Improving the ICU: part 1. Chest 2005;127:2151–64.Joint Commission on Accreditation of Healthcare Organizations has also identified hospital mortality of ICU patients as the core measure Available from: http://www.jcaho.org/pms/core_measures/icu_manual.htm. provide date of citationMcMillan TR, Hyzy RC. Bringing quality improvement into the intensive care unit. Crit Care Med 2007;35:S59–65.Norris C, Jacobs P, Rapoport J, Hamilton S. ICU and non-ICU cost per day. Can J Anaesth 1995;42:192–6.Goldfrad C, Rowan K. Consequences of discharges from intensive care at night. Lancet 2000;355:1138–42.Beck DH, McQuillan P, Smith GB. Waiting for the break of dawn? The effects of discharge time, discharge TISS scores and discharge facility on hospital mortality after intensive care. Intensive Care Med 2002;28:1287–93.Daly K, Beale R, Chang RW. Reduction in mortality after inappropriate early discharge from intensive care unit: logistic regression triage model. BMJ 2001;322:1274–6.Metnitz PG, Fieux F, Jordan B, Lang T, Moreno R, Le Gall JR. Critically ill patients readmitted to intensive care units: Lessons to learn? Intensive Care Med 2003;29:241–8.Rosenberg AL, Watts C. Patients readmitted to ICUs*: A systematic review of risk factors and outcomes. Chest 2000;118:492–502.Kerridge RK, Glasziou PP, Hillman KM. The use of “quality-adjusted life years” (QALYs) to evaluate treatment in intensive care. Anaesth Intensive Care 1995;23:322–31.Luce JM, Rubenfeld GD. Can health care costs be reduced by limiting intensive care at the end of life? Am J Respir Crit Care Med 2002;165:750–4.Atkinson S, Bihari D, Smithies M, Daly K, Mason R, McColl I. Identification of futility in intensive care. Lancet 1994;344:1203–6.Spielman B. Collective decisions about medical futility. J Law Med Ethics 1994;22:152–60.Luce JM, Alpers A. End-of-life care: what do American courts say? Crit Care Med 2001;29:N40–4.Knaus WA, Wagner DP, Zimmerman JE, Draper EA. Variations in mortality and length of stay in intensive care units. Ann Intern Med 1993;118:753–61.Critical Care Safety: Essentials for ICU PatientCare and Technology. Available from: http://www.ecri.org/criticalcare. provide date of citationStarfield B. Is US health really the best in the world? JAMA 2000;284:483–4.Kohn LT, Corrigan JM, Donaldson MS, editors. To err is human. Washington, DC: National Academies Press; 2000.Brennan TA, Leape LL, Laird NM, Hebert L, Localio AR, Lawthers AG, *et al*. Incidence of adverse events and negligence in hospitalized patients: results of the Harvard Medical Practice Study. N Engl J Med 1991;324:370–6.Zhan C, Miller MR. Excess length of stay, charges and mortality attributable to medical injuries during hospitalization. JAMA 2003;290:1868–74.Haywood RA, Hofer TP. Estimating hospital deaths due to medical errors. JAMA 2001;286:415–20.Tips to improve care in your ICU. Available from: http://www,sccm.org/tips. provide date of citationCritical Care Safety: Essentials for ICU Patient Care and Technology. Available from: http://www.ecri.org/criticalcare. provide date of citationChang SY, Multz AS, Hall JB. Critical care organization. Crit Care Clin 2005;21:43–53.Pronovost PJ, Angus DC, Dorman T, Robinson KA, Dremsizov TT, Young TL. Physician staffing patterns and clinical outcomes in critically ill patients: A systematic review. JAMA 2003;288:2151–2.Sample safety attitudes questionnaire from the University of Texas's (Houston) Center of Excellence for Patient Safety Research and Practice is available online. Available from: http://www.uth.tmc.edu/schools/med/imed/patient_safety/surveyandtools.htm. provide date of citationGarland A. Improving the ICU: Part 2. Chest 2005;127:2165–79.Quality in Critical Care -Beyond ‘Comprehensive Critical Care’ Quality Critical Care – recommended actions for Strategic Health Authorities (SHAs) Available at: http://www.dh.gov.uk/prod_consum_dh/groups/dh_digitalassets/@dh/@en/documents/digitalasset/dh_4123555.pdf.Centers for Disease Control and Prevention. National Center for Health Statistics. Deaths by place of death, age, race, and sex: United States, 1999-2002. Available from: http://www.cdc.gov/nchs/data/dvs/mortfinal2002_work309.pdf. [last accessed on 2002 Feb 21].Schuster MA, McGlynn EA, Brook RH. How good is the quality of health care in the United States? Milbank Q 1998;76:517–63.Chassin MR, Kosecoff J, Park RE, Winslow CM, Kahn KL, Merrick NJ, *et al*. Does inappropriate use explain geographic variations in the use of health care services? JAMA 1987;258:2533–7.McNeil BJ. Shattuck lecture: Hidden barriers to improvement in the quality of care. N Engl J Med 2001;345:1612– 20.Rapoport J, Teres D, Steingrub J, Higgins T, McGee W, Lemeshow S. Patient characteristics and ICU organizational factors that influence frequency of pulmonary artery catheterization. JAMA 2000;283:2559–67.Curtis JR, Cook DJ, Wall RJ, Angus DC, Bion J, Kacmarek R, *et al*. Intensive care unit quality improvement: A “how-to” guide for the interdisciplinary team. Crit Care Med 2006;34:211–18.Esteban A, Anzueto A, Frutos F, Alía I, Brochard L, Stewart TE, *et al*. Characteristics and outcomes in adult patients receiving mechanical ventilation: An international survey. JAMA 2002;287:345–55.Gallesio AO, Ceraso D, Palizas F. Improving quality in the intensive care unit setting. Crit Care Clin 2006;22:547–71.Berenholtz SM, Dorman T, Ngo K, Pronovost PJ. Qualitative reviewof intensive care unit quality indicators. J Crit Care 2002;17:1–12.Marik PE, Hedman L. What's in a day? Determining intensive care unit length of stay. Crit Care Med 2000;28:2090–3.Weissman C. Analyzing intensive care unit length of stay data: problems and possible solutions. Crit Care Med 1997;25:1594–600.Esmail R, Kirby A, Inkson T, Boiteau P. Quality improvement in the ICU. A Canadian perspective. J Crit Care 2005;20:74–8.Pronovost PJ, Angus DC, Dorman T, Robinson KA, Dremsizov TT, Young TL *et al*. Physician staffing patterns and clinical outcomes in critically ill patients: a systematic review. JAMA 2002;288:2151–62.The Leapfrog Group. Available from: http://www.leapfroggroup.org/about_us. [last accessed on 2006 Jan 12].

### 14.2: Quality Indicators in Critical Care: Patient Safety

**S K Todi**

### Introduction

“To err is human,” a seminal paper from Institute of Medicine (USA) in 1999, citing 44,000 to 98,000 deaths annually in USA due to medical errors, which is roughly equivalent to a jumbo jet full of passengers crashing every day, took the world by storm. This paper attracted huge media attention and gave rise to a new “safety” movement in medicine.

With increasing corporatization of the health sector in India, there is a growing demand from the consumers, regulatory authorities, and the government that healthcare providers adopt a culture of “safety” and hospital managers have taken this as the prime quality initiative. “Primum non norcere” – first, do no harm is being rediscovered and is the present day “mantra” in healthcare institutions all across the globe and to be competitive internationally, we need to firmly put this as the primary agenda of health care delivery in our country.

### Epidemiology

ICUs have been the main focus of delivering safe healthcare as the patient population are at greatest risk of harm here, due to multiple interventions, polypharmacy, increase workload, variability of staffing and patient related factors. Observers who attended ICU rounds found that staff reported a serious adverse event in 17% of patients. Self-reports and direct observations in a medical /surgical ICU found 1.7 errors per patient per day, one-third of these were potentially harmful. With an average length of ICU stay of three days, it turns out that every patient has a potential of serious error at least once during their ICU stay.

### Definition

The term “safety” is more diplomatic than “error” as the latter implies direct fault of healthcare provider. An error of “omission,” i.e. what we fail to do (meeting standard of care) is often termed as “quality” and error of “commission i.e. what has already been done (not meeting standard) is termed “safety”. Quality and safety are two sides of the same coin and it is difficult to know where quality ends and safety begins. Medical researchers have so far concentrated on clinical management part of patient care and only lately has attention been given towards research in implementation of therapy and safe patient care. In order to standardize and compare, regulatory authorities have laid down definitions pertaining to safe patient care.

**Patient Safety:** It is defined as the absence of the potential for, or occurrence of, healthcare–associated injury to patients. It is created by avoiding medical errors as well as taking action to prevent errors from causing injury.

**Error:** It is defined as mistakes made in the process of care that result in, or have the potential to result in, harm to patients. Mistakes include the failure of a planned action to be completed as intended or the use of a wrong plan to achieve an aim. These can be the result of an action that is taken (error or commission) or an action that is not taken (error of omission).

**Incident:** Unexpected or unanticipated events or circumstances not consistent with the routine care of a particular patient, which could have, or did lead to, an unintended or unnecessary harm to a person, or a complaint, loss, or damage.

**Near Miss:** An occurrence of an error that did not result in harm.

**Adverse Event:** An injury resulting from a medical intervention.

**Preventable Adverse Event:** Harm that could be avoided through reasonable planning or proper execution of an action.

### Measurement

As patient safety is a concept and an abstract term, converting it into numerical terms for research and audit purposes is difficult. One also has to consider many dimensions of safe patient care. We all try to practice safe patient care but when it comes to quantifying it, certain basic principles need to be followed.

Principles of management from industry are being increasingly incorporated in medicine and this is most evident in regards to patient safety. Safe industries (e.g. aviation) report defect rate in terms of sigma or defects Per 10,000 or 1,000 events. One sigma equates to a 69% defect rate and six sigma equals three defects per million. Healthcare industry's record is abysmal in this regard which runs at one or two sigmas.

Any quantification tool will be meaningful if it consists of a numerator (number of events observed) and denominator (number at risk) so that a rate can be calculated. It is labor intensive to keep a tab on rates of adverse events, and a more subjective approach may be appropriate some time, which acts to highlight problem areas to be specifically addressed in a more objective way.

Examples of such a subjective approach will be peer review, morbidity and mortality conferences, investigation of liability claims, and incident reports. In all these, a single event is analyzed, which is not linked to a denominator which limits the ability to estimate rates. Nonetheless, they help to identify problem areas.

**Incident Report:** It evaluates how a single patient is harmed but can also be utilized to look at near misses i.e. incidents that did not but could have caused harm. The ICUSRS project pioneered by Dr. Pronovost from Johns Hopkins is an example of such incident reporting system which is web based.

To be successful, an incident reporting system should be voluntary, anonymous, and not linked with any form of punitive measure. The ICUSRS system is open to participating hospitals and personnel can enter incidents and near misses confidentially, which is analyzed centrally and feedback is given. Over 1700 reports have been analyzed in the system. In order to standardize reporting systems, hospitals are encouraged to report incidents in terms of patient variables, exposure variables and outcome variables. A framework for evaluating such reports is also laid down, which analyses the incident reports under Patient factor, Provider factor, Team factor, Task factor, Training and Education factor, ICU environment and Institutional environment.

**Root Cause Analysis**- This is a more focused enquiry on certain incidents which are deemed to be important for patient safety. A sentinel event is identified, important preventive aspects of this event are discussed by the “safety” team and the safeguards are implemented.

**Failure Mode and Effects Analysis (FMEA)** -Both the incident report and root cause analysis are post hoc analysis which tries to improve patient safety after the incident has occurred. A more proactive approach where a problem area is identified prospectively, and all possible preventable aspects are discussed and remediable measures are taken. This approach takes away the primary burden from an individual and focuses more on system failure. In an FMEA, an error-prone process is identified and a multi-disciplinary team is formed to analyze the process from multiple perspectives. The team systematically assesses failure modes and the urgency with which each failure mode should be addressed. Where RCA can be thought of as an expanding circle of inquiry that is focused on a sentinel event, FMEA is a linear process that examines a selected process from start to finish. Conducting FMEA is highly time consuming and labor intensive, so its use should be restricted to areas prone to serious adverse events. Regulatory authorities are now making it mandatory in USA and UK for medical and nursing directors of ICUs to conduct at least one FEMA annually.

### Implementation

Implementing “safety” culture in the ICU has to come from a strong leadership primarily from the ICU director, backed by a willing management.

The first step in our country is to ensure that the healthcare providers are assured that no punitive actions will be taken against them if an adverse event is identified or reported. In fact some institutions in India have started rewarding such bold steps of revealing errors to the authority. The concept of ‘system failure’ rather than “individual failure” needs to be enforced.

Secondly, a system of reporting adverse events has to be in place for audit or root cause analysis. This system should be discreet and could be paper or computer based.

Thirdly, an audit of incident report, root cause analysis or FMEA should be performed periodically by a multidisciplinary team consisting of ICU director, Nursing director, Quality control personnel, and hospital administrator. Corrective measures should be identified and feedback given to healthcare providers.

Finally, established practices for decreasing errors like computerized physician order entry (CPOE) system, patient identification tags, check list for blood transfusion should be in place and checked periodically for compliance.

There is a great need for research in this field in our country to identify areas of vulnerability, and finding cost effective solutions to problems of patient safety.

### Further reading

Rothschild JM, Landrigan CP, Cronin JW, Kaushal R, Lockley SW, Burdick E, *et al*. The Critical Care Safety Study: The incidence and nature of adverse events and serious medical errors in intensive care. Crit Care Med 2005;33:1694–700.Duwe B, Fuchs BD, Hansen-Flaschen J. Failure mode and effects analysis application to critical care medicine. Crit Care Clin 2005;21:21–30.Pronovost PJ, Thompson DA, Holzmueller CG, Lubomski LH, Morlock LL. Defining and measuring patient safety. Crit Care Clin 2005;21:1–19.Berenholtz SM, Pronovost PJ. Monitoring Patient Safety. Crit Care Clin 2007;23:659–73.Bria WF 2nd, Shabot MM. The electronic medical record, safety, and critical care. Crit Care Clin 2005;21:55–79.Render ML, Hirschhorn L. An irreplaceable safety culture. Crit Care Clin 2005;21:31–41.Hussain E, Kao E. Medication safety and transfusion errors in the ICU and beyond. Crit Care Clin 2005;21:91–110.Heffner JE, Ellis R, Zeno B. Safety in training and learning in the intensive care unit. Crit Care Clin 2005;21:129–48.

### 14.3 Quality indicators in Critical Care: Personnel Development

**Suresh Ramasubban**

Historically, the specialty of critical care started with cohorting of acutely ill patients into separate clinical areas. These discrete geographical areas were subsequently named Intensive care units. With the advent of various new technologies, these specialized clinical areas became segregated from other hospital wards and had personnel needs which were different from that of the other hospital wards.

The delivery of care in the ICUs requires the presence of highly trained, skilled and motivated personnel who can apply modern techniques and interventions in an appropriate fashion to provide the highest quality of care. Irrespective of the type of ICUs and their geographic locations, all ICUs have the responsibility to provide services and personnel that ensure quality care to patients. With the Leapfrog initiative in the US based on the Institute of Medicine's (IOM) report, commitment to high quality care is now of paramount importance. Personnel Development is an integral part of this quality initiative. This chapter will focus on ICU personnel development and quality initiatives focusing to personnel development. This will be discussed under the following headings:
Definition of Staff in ICUTraining requirementStaffing logisticsQuality measures

### Staff in ICU

Irrespective of the model of critical care delivery in an ICU, a multidisciplinary approach is recommended by the SCCM. As an example, medication errors are reduced significantly in hospital with intensivist staffing and multidisciplinary rounds. This involves the presence of dedicated ICU personnel, especially the intensivist, ICU nurse, ICU pharmacist and respiratory therapist.

Intensivist is a physician who is trained in a primary specialty such as Medicine/Anesthesia/Surgery/Chest Medicine) and has a certificate of special qualification in critical care. He diagnoses, manages, monitors, intervenes, arbitrates and individualizes the care to each patient at risk, in the midst of or recovering from critical illness. He/she should be immediately and physically available to patients in the ICU. The credentials should include both cognitive and procedural skills.

ICU House staff members are either physicians in training or otherwise who are fully dedicated to the ICU and have no other responsibility and are on site to provide all emergency care to the patient.

An ICU nurse should be a licensed nurse with preferably added certification in critical care. Although certification is not mandatory, certification validates to patients and employers that a nurse is qualified and has gone through rigorous training requirements to achieve the additional credential.

ICU pharmacist is defined as a practitioner who is a qualified pharmacist and has specialized training or practice experience providing pharmaceutical care for the critically ill patient.

The presence of an ICU pharmacist as part of the ICU team improves quality of care in the ICU by reducing medication error by as much as 66%.

Another important personnel of the ICU is the respiratory therapist who provides cardio-respiratory care to critically ill patients. The absence of trained therapist should lead to establishment of training programs for Respiratory therapist

### Training

Training is must for maintaining and further up gradating of skills of the ICU personnel. Imparting training based on the identified need is essential for any sensible unit. In the absence of any certification, nurses working in the ICU should have periodic assessment of competence by the nursing director with provision for feedback and need based education curriculum. This competency assessment should be standardized according to nursing guidelines of AACN.

House staff should have had training in advanced airway management and ACLS. FCCS/BASIC critical course training is recommended but not mandatory for ICU House staff.

### Staffing Logistics

ICU staffing pattern can be classified as low intensity (no intensivist or elective intensivist consultation) or high-intensity (mandatory intensivist consultation or closed ICU (all care directed by intensivist) groups. High-intensity staffing is associated with lower hospital mortality, lower ICU mortality, reduced hospital LOS, and reduced ICU LOS.

The lack of adequate staffing of nurses leads to delays in weaning patients, higher infection rates, increased readmission rates, increased medication errors and increased length of stay. Excessive nursing workload as defined by “hours per patient days” or “nurse/patient ratios” is associated with increased mortality in critically ill patients. Staffing pattern for nurses should take into account patient load and case mix. The gold standard for staffing should be one nurse for each critically ill patient.

Inadequate house staff leads to poor emergency care and poor continuity of care; adequate staffing pattern should be taking into account patient load and acuity of care.

### Quality Measures

Quality measures in the ICU are predominantly medical outcomes related but since the ICU provides service to relatives and friends, ICU personnel, the hospital and the society, other parameters must also be used. These include economic outcomes, psychosocial and ethical outcomes and Institutional outcomes.

Institutional outcomes like staff satisfaction and turnover rate are important measure of quality in the ICU related to personnel. Higher rates of staff turnover leads to increased costs, increased training time, decreased morale and increased stress on remaining staff, leading to decreased quality of performance and worse patient outcomes.

Each ICU should measure and control regularly the efficiency of the use of nursing manpower evaluating the work utilization ratio (WUR) by recommended scoring tools. Measuring staff satisfaction is an important quality initiative. Staff retention rates should be obtained from personnel records and data of job satisfaction should be obtained from questionnaires or exit interviews. Days or hours of training should be monitored to ensure quality of personnel development

**Conclusion:** A multidisciplinary approach with adequate ICU personnel and staffing pattern combined with ongoing training and need based skill development and measurement of institutional outcomes is necessary to provide quality critical care.

**Further Reading**

Brilli RJ, Spevetz A, Branson RD, Campbell GM, Cohen H, Dasta JF, *et al*. Critical care delivery in the intensive care unit: Defining clinical roles and the best practice model. Crit Care Med 2001;29:2007–19.Haupt MT, Bekes CE, Brilli RJ, Carl LC, Gray AW, Jastremski MS, *et al*. Guidelines on critical care services and personnel: Recommendations based on a system of categorization of three levels of care. Crit Care Med 2003;31:2677–83.European Federation of Critical Care Nursing Associations, EfCCNa Position Statement on workforce requirements within European Critical Care Nursing. 2007.Tips to improve care in your ICU. Available from: http://www,saccm.org/tips.Quality in Critical Care -Beyond ‘Comprehensive Critical Care’ Quality Critical Care - recommended actions for Strategic Health Authorities (SHAs).Pronovost PJ, Angus DC, Dorman T, Robinson KA, Dremsizov TT, Young TL. Physician staffing patterns and clinical outcomes in critically ill patients: A systematic review. JAMA 2003;288:2151–62.Garland A: Improving the ICU: Part 1 and 2. Chest 2005;127:2165–79.ISCCM Guidelines: Proposed Guidelines for ICU's in India.ISCCM Guidelines: critical care delivery in intensive care units in India.

### 14.4 Quality Indicators for ICU: Process Parameters

**George John, N Ramakrishnan**

The Critical Care Services in a modern hospital has a vital role to play in delivering prompt, appropriate and adequate care to acutely ill patients. Acutely ill patients can present with a variety of pathophysiological derangements which need rapid repeated interventions with constant monitoring and further interventions based on the results of the monitoring process. These interventions involve multiple components – all of which need to be seamlessly integrated to optimize outcome. In a study in which engineers observed patient care in ICUs for twenty four hours periods, it was found that the average ICU patient required ***178 individual interactions per day***. These included a range of interventions from physical maneuvers (such as positioning the patient) to medication administration.

**Quality and safety** are two facets of a system designed to deliver optimum care. The terms have been separated as two components by defining ***quality*** as referring to errors of omission and s***afety*** as errors of commission. Quality of care is an important issue because ***the cost of non-quality in any enterprise is more expensive than investing in quality.*** Quality of Care is defined as the degree of correspondence between goals set and goals achieved in relation to patient care without excessive use of financial resources. *Hence, quality is the **ratio** of standard achieved / expected standard.* It is 1.0 if all standards are achieved.

**Quality of care** is a complex process that can be monitored on three levels:
**Structure:** This includes architectural design, staffing, nurse: patient ratio, bed occupancy and all other components of structure related to quality.**Process** refers to the current practice of care delivery, hand washing and implementation of other guidelines.**Outcome:** Indicators of outcome such as nosocomial infection rates, mortality stratified to severity of illness and other outcome measures are the most valuable and readily recognised indicators of quality.

### Quality in Processes

Critical care services should employ best evidence practices, such as those described in ‘care bundles’.Patients requiring critical care are entitled to the care given by dedicated, highly skilled, multidisciplinary teams.Critical illness has a great impact on the lives of patients and their families. Decisions about care should be made in partnership between the critical care team, the patient, and relatives.Continuity of care and facilities are important throughout the patient's care period but especially when stepping down to lower levels of care, to general wards or home.

The 20 fundamental quality indicators for critical care developed by the Spanish Society of Intensive and Critical Care and Coronary Units (SEMICYUC) are:
Compliance with hand hygiene protocolsProviding information to families of patients in the ICUAppropriate sedationAppropriate pain managementEGDT in sepsisEarly enteral nutritionProphylaxis for GI bleed in those undergoing invasive mechanical ventilationInappropriate transfusion of packed cellsSemirecumbent position for patients on invasive mechanical ventilationVentilator associated pneumoniaPrevention of thromboembolismEarly administration of acetyl salicylic acid in acute coronary syndromeEarly reperfusion therapy in STEMIMonitoring ICP in severe traumatic brain injury with CT findingsSurgical intervention in traumatic brain injury with subdural and/or epidural hematomaProtocols and implementation of withholding / withdrawing life supportOrgan donationPerceived Quality Survey at discharge from ICUPresence of an intensivist in the ICUMaintaining an adverse events register

### Safety - Critical Incidents

Errors increase as a function of complexity. ***Adverse events*** are defined as those which have resulted in actual harm to the patient due to the management of a disease process and not as a result of the underlying disease. A ***near miss*** is an unplanned event that does not result in injury, illness, or damage; but has the potential to do so. Only a fortuitous break in the chain of events prevents an injury, fatality or damage. Near misses do not necessarily result in patient harm (but has the potential to do so). Analyzing near misses has the advantage of gaining insight into unsafe practices and to discover reasons why it did not lead to an adverse event. Consider an iceberg where 90% is submerged under water. Fatal adverse events are only the tip of the iceberg. Major adverse events comprise the visible portion below the tip. The submerged section is divided into two layers. The minor adverse events are just below the surface of water and the bottom layer is the near misses.



More than 60 years ago, Heinrich proposed a 300-29-1 ratio between near-miss incidents, minor injuries, and major injuries. Heinrich also estimated that 88% of all near misses and workplace injuries resulted from unsafe acts. Interestingly, the 300-30 ratio of near misses to injuries is referred to as a “law,” when in fact it was only an estimate. More than 30 years later, this “law” was actually tested empirically. Frank E. Bird, Jr. analyzed 1,753,498 “accidents” reported by 297 companies. The result was a new ratio: For every 600 near misses, there were 30 property damage incidents, 10 minor injuries, and one major injury. It's likely the base number is much larger than 600. A reduction of near misses (events at the bottom of the iceberg) should lead to a reduction in the number of events at the exposed top of the iceberg.

Unlike other areas in medical care, adverse events can occur even when care is delivered appropriately; as an example renal failure can occur even with appropriate dosing of aminoglycosides. Crude event rates do not provide an adequate measure of adverse events as it does not account for the unknown range of opportunities for harm.

#### Error Detection

Clinicians make decisions in a highly complex environment by negotiations and compromises as they trade-off between competing goals. In order to characterize the systemic causes of error in such environments, we need to identify the pressures (e.g. fatigue, workload, policy, and lack of resources) that push people towards these boundaries, and then make efforts to counteract pressures.

#### Error Resilience

A realistic approach is to recognize that human error cannot be eradicated, but that the negative consequences of an error can be controlled. Thus, an error resilient system should have the following targets:
Control the propagation of human error towards accident occurrenceReduce adverse eventsCorrect / Recover from error

Error correction forms an integral part of the cognitive system underlying critical care (and other complex tasks). In keeping with contemporary human error research, approaches seeking to eradicate error fail to recognize that error recovery is integral to any cognitive work. The critical role of error recovery mechanisms in the maintenance of system safety is neglected by approaches that focus exclusively on completed errors.

### Tools to Deliver Quality Medical Care

#### Evidence based medicine

Measures to improve quality of healthcare delivery and patient safety must be based on evidence. However, unlike the rest of medical care, there has been a push to prioritizing action over evidence in this field. It is essential to ensure that measures to improve patient safety and quality of care do so. Rigorous evaluation of such measures does not necessarily mean that randomized trials are always needed. Robust evidence can be obtained by alternative strategies such as “before and after” studies with concurrent control groups and time series designs with measurable outcomes. Such an approach will ensure that the solutions implemented will not squander resources or blind us to the adverse effects of interventions.

#### Protocols, checklists, bundles and guidelines

Delivery of healthcare is a science in three domains: the first is to understand disease biology/ dynamics; the second is to find effective interventions; the third is to find strategies to deliver the most appropriate intervention effectively by incorporating relevant research findings into daily practice. The inability to translate top quality research into medical practice is a major problem in healthcare. Published best practice guidelines do not by themselves reliably improve patient care. Continuing educational programs, use of quality indicators and feedbacks are important elements of the strategy to deliver the best evidence based care to the bedside. ***Checklists*** have been found to be effective in implementing evidence based management bundles. They help in two ways: with memory recall and with making explicit the minimum expected steps in complex processes. An average ICU patient requires multiple individual interventions per day and checklists help in establishing higher standards of baseline performance. Even the simple strategy of having doctors/ nurses make their own checklists for what they thought should be done each day improves consistency and quality of care.

#### Clinical Management Bundles:

There are more opportunities for clinicians to modify their care in an effort to improve patient outcome as more high-level evidence in critical care medicine becomes increasingly available. Recent examples of some of the evidence that should have triggered reevaluation of clinicians' approaches to patients include the use of steroids in septic shock, early goal-oriented resuscitation in sepsis, hypothermia for out of hospital cardiac arrests, tight blood glucose control, use of spontaneous breathing trials, and lung-protective ventilation. In these scenarios, it is tempting to locally implement the exact protocol used in the study. As with any change initiative, this implementation process can be complex in order to improve the delivery of scientifically proven therapies, the concept of “bundles” is useful.

A “bundle” is a group of interventions related to a disease process that, when executed together, result in better outcomes than when implemented individually. The science behind the bundle is so well established that it should be considered standard of care. Bundle elements should be dichotomous and compliance should be measurable as yes/no answers. Bundles avoid the piecemeal application of proven therapies in favor of an “all or none” approach. This strategy provides a simple but rigorous check list and documentation. It facilitates easy performance monitoring.

#### 1. Basic Bundle for all ICU patients: A checklist

##### Remember: Fast hug

Feed, Analgesia, Sedation, Throboprophylaxis, Head of bed elevation, Ulcer prophylaxis, Glucose Control

#### 2. Sepsis Resuscitation Bundle

Serum lactate to be measured

Blood cultures obtained prior to antibiotic administration

From the time of presentation, broad-spectrum antibiotics administered within 3 hours for Emergency Department admissions and 1 hour for non-ED ICU admissions

##### In the event of hypotension and/or lactate > 4 mmol/L (36 mg/dl)

Deliver an initial minimum of 20ml / kg of crystalloid (or colloid equivalent).

Use vasopressors for hypotension not responding to initial fluid resuscitation to maintain mean arterial pressure (MAP) > 65 mm Hg

##### In the event of persistent hypotension despite fluid resuscitation (septic shock) and/or lactate > 4 mmol/L (36 mg/dl)

Achieve central venous pressure of 8mm Hg

Achieve central venous oxygen saturation of > 70%

#### 3. Sepsis Management Bundle

Low dose steroid administered for septic shock in accordance with a standardized ICU policyDotrecogin alfa (activated) administered in accordance with a standardized ICU policyGlucose control maintained > lower limit of normal, but < 150 mg/dl (8.3 mmol/L)Inspiratory plateau pressure maintained < 30 cm H2O for mechanically ventilated patients

#### 4. The ‘Antibiotic Care Bundle’

Clinical criteria for initiation of antimicrobial therapyActively get specimens for microbiologyInitial empiric antibiotic choice based on local policyRemove infected source: foreign body, drain collectionsModify when microbiology results are availableDaily review of antibiotic choice and continuationRegular expert input

#### 5. Ventilator Care Bundle

##### a. General:

DVT prophylaxis: Unfractionated heparin 5000 units every eight hourly or twice daily

GI stress ulcer prophylaxis: H2 blocker as prophylaxis

Eye and Skin Care

##### b. Skin prep

2% w/v chlorhexidine is better than 10% w/v povidone; chlorhexidine povidone and chlorhexodine sequential cleaning is even better as skin preparation for central line insertion.

Chlorhexidine % v/v is equivalent to only 1/5 of w/v solution. Chlorhexidine 2.5 % v/v is equivalent to only a 0.5% w/v solution - inadequate for skin preparation, but adequate for hand hygiene

##### c. Maintain internal environment

Hb ≥ 7g%;

Electrolytes

Glycemic control

##### d. Support of failing organ systems as appropriate: inotropes, dialysis

##### e. Infection control

##### Hand Hygiene

Use 60 – 90% alcohol or 0.5-1.0% chlorhexidine (w/v)

Airway - orotracheal

Oral Hygiene – chlorhexidine 2% or povidone 10% at least thrice a day

Ventilator Circuits – change if visibly contaminated

Suction - no difference between closed and open

Body Position – 30° – 45° Head of Bed up (not just the head of patent as a sedated patient will slip down)

##### f. Oxygenation and Ventilation settings

Lowest FiO2 and adequate PEEP- to keep PaO2 55 – 80mm Hg

Mode: Non Invasive if possible

Volume Control mode:

- Tidal Volume 6ml / kg

- High rate if CO2 high – upto 35 / minute; permissive hypercapnoea

I - E ratio 1:1 to 1:3

- If pH < 7.30 - use HCO3 infusion

Measures to decrease CO2 production (sedation, decrease temperature)

Intermittent interruption of sedation if there is 1:1 nursing care

#### 6. Bundle for prevention of Ventilator Associated Pneumonia is known as WHAP

Early **W**eaning

**H**and Hygiene

**A**spiration Precautions

**P**revention of contamination

### Caution regarding bundles!

First, it cannot be emphasized adequately that the *time-consuming process of protocol implementation could negatively impact the acquisition and maintenance of high-level clinical skills. In other words, the protocol can become a priority and patient care can become uncoupled from skillful clinical decision making. Clinicians should always be aware that when implementing an evidence-based approach, the importance of being good clinicians should always be kept in mind.*

Second, the development of bundles is also potentially vulnerable to manipulation for inappropriate ends. Seeing in these bundles a potentially powerful vehicle for promoting their products, some pharmaceutical and medical-device companies have begun to invest in influencing the adoption of guidelines that serve their own financial goals. There is thus a question of whether these bundles are “evidence based” or “evidence biased”. The relationship between scientific societies and industry is complex and fraught with problems. Theoretically, each group exists to improve patient care and outcome. In practice, the primary objective of any industry is to sell its products and make a profit while the scientific society exists to represent its members, to impartially judge available evidence and provide advice and support to its members in the best interest of patient care. The process of developing guidelines should not be perceived as a marketing vehicle for any particular industry.

**Further Reading**

Donchin Y, Gopher D, Olin M, Badihi Y, Biesky M, Sprung CL, *et al*. A look into the nature and causes of human error in the intensive care unit. Qual Saf Health Care 2003;12:143–7.Leape LL, Berwick DM. Five years after To Err Is Human What have we learned. JAMA 2005;293:2384–90.Garland A. Improving the ICU: Part 1. Chest 2005;127:2151–64.Garland A. Improving the ICU: Part 2. Chest 2005;127:2165–79Available from: http://www.semicyuc.org/calidad/quality_indicators_SEMICYUC2006.pdf.Gawande A. The Checklist. The New Yorker. Available from: http://www.newyorker.com/reporting/2007/12/10/071210fa_fact_gawande. [last assessed on 2007 Dec 10].Auerbach AD, Landefeld CS, Shojania KG. The tension between needing to improve care and knowing how to do it. N Engl J Med 2007;357:608–13.Scales DC, Laupacis A. Health technology assessment in critical care. Intensive Care Med 2007;33:2183–91.Ranganathan P. Cost of ICU care in India - Prohibitive or Justified? In: Nayyar V, Peter JV, Kishen R, Srinivas S, editors. Critical Care Update 2007. New Delhi: Jaypee Brothers Medical Publishers (P) Ltd; 2008. p. 20–7.Dastur FD. Quality and safety in Indian Hospitals. J Assoc Phys India 2008;56:85-7.Najjar-Pellet J, Jonquet O, Jambou P, Fabry J. Quality assessment in intensive care units: proposal for a scoring system in terms of structure and process. Intensive Care Med 2008;34:278–85.Weinert CR. The science of implementation: changing the practice of critical care. Curr Opin Crit Care 2008;14:460–5.Patel V, Cohen T. New perspectives on error in critical care. Curr Opin Crit Care 2008;14:456-9.MacLaren R, Bond CA, Martin SJ, Fike D. Clinical and economic outcomes of involving pharmacists in the direct care of critically ill patients with infections. Crit Care Med 2008;36:3184–9.Gallesio AO. Improving quality and safety in the ICU: a challenge for the next years. Curr Opin Crit Care 2008;14:700–7.Valentin A. Reducing the number of adverse events in intensive care units. Controversies in Intensive Care Medicine. ESICM publication; 2008. p. 337–42.

#### 14.5 Quality Indicators in Critical Care – Outcome Parameters

**George John, N. Ramakrishnan**

**The cost of non-quality in any enterprise is more expensive than investing in quality.**

The difference is magnified in settings such as the ICU, where the baseline costs are among the highest in the health care domain.

In order to choose *outcome* parameters in any enterprise, the mission goals must be clearly defined. In the critical care setting, the goals are as follows:
To preserve meaningful life: In this context “meaningful life” refers to a quality of life valued by the patient.To provide specialized care to patients in order to sustain, protect and rehabilitate them during their treatment for a critical illness or injury: “Specialized care” implies care in an environment where it is possible to provide real time monitoring of vital parameters along with the ability to intervene rapidly when necessary.To provide compassionate palliative care to those who are dying from irreversible diseases in order to alleviate suffering during their final hours.To ensure the viability and sustainability (economic and human resources) of the unit in order to deliver the above modes of care professionally as a team.

Sentinel events are measurable events which indicate the achievement (or non achievement) of a goal. The sentinel events in the corresponding domains would be as follows:

### Preservation of Life

The marker of a negative outcome in this domain would be ***mortality***. The mortality can be measured as:

***Crude mortality:*** Crude mortality rates *cannot* be used to measure quality of ICU care because they do not adjust for differences in diagnosis and severity of disease.

***Standardized Mortality Ratio:*** Disease based/Severity Score adjusted: The Standardized Mortality Ratio (SMR) is defined as the *ratio of the observed mortality rate to the expected mortality rate*. This permits performance-based comparisons of ICUs by adjusting for disease category and severity of physiological derangement. The reference values for the expected mortality rates are obtained by documenting mortality rates of patients from a large number of ICUs in a specific population. These are then stratified based on disease categories and within multiple outcome score bands of standard ICU scoring systems. If the SMR for an ICU is <1, then the outcomes for that unit are interpreted to be better than the overall outcomes of the reference set used to develop the scoring system. Alternatively, an SMR of >1 signifies that the observed mortality rate is higher than the expected mortality rate, suggesting that the quality of care needs to be improved.

### Specialized Care During Critical Illness

The markers for this domain would be:
Morbidity and post discharge events

***Morbidity:*** Morbidity could be due to three broad reasons: *chance, faults in the system or human error.* System faults and human errors are appropriate targets for quality improvement. Faults in the system include overutilization, underutilization and misutilization of resources.

Measures of morbidity during ICU stay are:

**Table d32e4631:** 

Resource utilization & availability	General Complications:
New admissions, Patient	*Rates for:*
Days, Ventilator Days	Airway Tube block (endotracheal, tracheostomy)
Device Days (airway, CVC, NG tube, urinary)	Reintubation, Unplanned extubation
Nurse: Patient ratio	
Doctor: Patient ratio	
Non availability of ICU bed (denied request)	
Non availability of Ventilator (denied request)	
Length of Stay	
Unplanned Readmission	
Rate	
Equipment downtime	

**Table d32e4688:** 

Infection Related Complications:	Medication / Transfusion Related:
Hand hygiene compliance	Medication Errors
Nosocomial Infection Rate	Prescription of a wrong medication
CRBSI	
VAP	Inadequate prescription – wrong dose, time schedule
Wound / Soft tissue	
Urinary	
Others – Para nasal sinus, Eye	Wrong administration – dilution solution, strength, infusion rate
MDR Infections: ESBL, MRSA	Adverse Drug Reaction Blood / Component transfusion reaction
Fungal Infections	
Antibiotic De-escalation rate	

### Palliative Care

The sentinel events to track are:
Futility: number of patients being admitted for futile care to the ICU; there is, as yet, no universally accepted definition of futility.Number of counseling sessions for family membersFamily satisfaction

### Viability and Sustainability of the ICU as a Healing Unit

It is obvious that intensive care is based on team work and the markers of quality should not be restricted to a reductionist view of intensive care. The following are important:

#### a. Economic Issues:

This is important in our Indian setting where the public funding for the tertiary level of care is inadequate, the level of health insurance cover is low and health care bills drive families into debt.

It has to be looked at from the

Health providers viewpoint - capital / running expenses versus incomePatients'/ Families' viewpoint - cost per person who has not survived; debt the family / person has incurred per person alive / expired. This is not an issue covered in Western literature and we need to have our own data.

#### b. Education and Safety issues

Patient safety: error detection, reporting and error resilience;Staff safety: needle stick injuries; HBsAg immunization rate in ICU personnel; HIV prophylaxis given; staff Burn-outEducation and Training: Personnel trained – medical, nursing, technical; continuing medical, nursing and technical education

### Errors in Medical Practice

It is very important to have a pragmatic perspective of error in the ICU. Errors increase as a function of complexity. In a study in which engineers observed patient care in ICUs for twenty four hour periods, the average ICU patient required ***178 individual interactions per day***. These included a range of interventions from physical maneuvers (positioning the patient, physiotherapy) to medication administration.

#### Error Detection

Clinicians in the ICU make decisions in a highly complex environment by negotiations and compromises as they trade off between competing goals. In order to characterize the systemic causes of error in such environments, we need to identify the pressures (e.g. fatigue, workload, policy and / or lack of resources) that push people towards these boundaries and then make efforts to counteract these pressures.

The phrase ***‘error in evolution’*** denotes the progression of a series of small mistakes towards a cumulative adverse event. Erroneous decisions undergo a selection process based on their anticipated consequences. The figure below illustrates the progression of error in critical care, where personnel (clinicians, nurses, technicians and others) conducting routine work hit a boundary and where they come close to making an error (***near miss***). “Near miss” is a breach of the first boundary and is a violation of the bounds of ***safe practice***. At this stage, the error can still be detected and corrected before the second boundary is crossed. This is a window of opportunity to detect and prevent a potentially adverse event. If only adverse events are reported, the “near misses” will continue to remain undetected. Since “near misses” are an integral component of the chain of events leading to an adverse event, detection, reporting and reducing these should be an integral component of any strategy to reduce errors in any system.

If undetected / uncorrected, it can proceed to the next stage - an ***adverse event*** which occurs when the second boundary is crossed.



The traditional culture in Medicine pins errors on to individuals. In reality, errors occur because there are multiple “mini-errors” distributed across non living objects (monitoring, hand over notes, computers etc.) as well as in the minds of technicians, nurses and clinicians. *It is essential to internalize the perspective that faulty action is a product of flawed thinking across the system - this is the concept of **“distributed cognition”**.* The perspective that distributed cognition is responsible for any error shifts the focus of analysis from the study of individuals in controlled settings to the study of groups of individuals in their real-world context. Using this framework, a collective workflow can be reconstructed from events of critical importance that are spatially or temporally correlated. This mode of analysis focuses on the identification of *vulnerabilities and flaws in the system* (as opposed to the action of a single individual). In contrast, retrospective analysis of individual error is vulnerable to the bias of 20/20 hindsight: ***actions leading to the error may be viewed as incorrect although they may have been the best alternative with the information available at that point in time.***

The cognitive and emotional demands imposed by multitasking, interruptions and handovers during change of shift can be sources of error. Gaps in information flow occur during handovers. *Error production and error detection rate are studied as a function of task demand.* Three levels of demand are considered; high, average, low work load. While it might seem intuitive that more errors would occur at a high workload, the actual results of research show an apparent paradox: ***the greatest number of errors occurred at a low workload, with the least number of errors at a high workload. However, at high workload, error detection was reduced, leading to a much higher rate of adverse events. The rate of error detection improves with practice.***

#### Error Resilience

A realistic approach is to recognize that error cannot be completely eliminated, but that the negative consequences of an error can be controlled. Thus, an error resilient system should have the following targets:
control the propagation of error towards occurrence of adverse eventsredmuce adverse eventshave a strategy for error correction / recovery

Error correction and recovery should form an integral part of the cognitive system underlying quality in critical care. The critical role of error resilience in the maintenance of safety in any system is neglected by approaches that focus exclusively on completed errors.

Research findings challenge the common perception that experts are somehow infallible. They are consistent with error research in other domains which show a constant rate of error regardless of expertise (with the exception of absolute beginners who make significantly more errors at the beginning of their learning curve). ***Clinicians at all levels of expertise make errors; however, experts make errors from which it is easier to recover.***

**The cost of non-quality in any enterprise is more expensive than investing in quality.**

**Further Readings**

Donchin Y, Gopher D, Olin M, Badihi Y, Biesky Y, Sprung CL, Pizov R, Cotel S. A look into the nature and causes of human error in the intensive care unit. Qual Saf Health Care 2003;12:143–7.Leape LL, Berwick DM. Five years after To Err Is Human What have we learned. JAMA 2005;293:2384–90.Weinert CR. The science of implementation: changing the practice of critical care. Curr Opin Crit Care 2008;14:460–5.Patel V, Cohen T. New perspectives on error in critical care. Current Opin Crit Care 2008;14:456–9.MacLaren R, Bond CA, Martin SJ, Fike D. Clinical and economic outcomes of involving pharmacists in the direct care of critically ill patients with infections. Crit Care Med 2008;36:3184–9.Gallesio AO. Improving quality and safety in the ICU: a challenge for the next years. Curr Opin Crit Care 2008;14:700–7.

#### 14.6 Quality Indicators: Infection Control

**D P Samaddar**

Value addition in health-care is directly proportional to quality and inversely proportional to cost. The goal, therefore, should be to obtain the highest quality healthcare at an affordable price. Hospital acquired infection or healthcare related infection (HAI) creates an imbalance between quality and cost by increasing mortality, morbidity, length of stay, psychological stress and disproportionately higher financial drain. This imbalance can be bridged by improvement in the process and system as quality of healthcare is progressively being linked to process and system and not to individuals. Compliance to processes and its qualitative impact on the delivered healthcare can be assessed by selecting and monitoring appropriate quality indicators.[1]

### 1. Magnitude of Problems

Healthcare-associated infections (HAIs) account for an estimated 1.7 million infections, 99,000 deaths, and $4.5 billion in excess healthcare costs annually in USA.[2] In England, the cost incurred due to HAI is estimated to be 3.6 million pounds per year per health unit.[3]

Bloodstream infection (BSI) alone causes an estimated 26,250 deaths per year. It is ranked the eighth leading cause of death in the United States.[4] Attributable death due to ventilator-associated pneumonia (VAP) is 15 to 30 % with overall mortality of 42 %.[3] HAI also causes increased length of stay, nonavailability of beds due to unacceptable bed occupancy. The reported average prolonged stay for urinary infection is 3.8 days, 7.4 days for surgical-site infection, 5.9 days for pneumonia and seven to 24 days for primary bloodstream infection.[3]

The emergence and spread of antimicrobial-resistant organisms is a major concern. HAI also is an important issue because 30 to 50 % reduction in HAI is possible by running an efficient infection control program.[3]

### 2. Objective

Reduction in the incidence of nosocomial infection is the main objective but it is a broad based and less specific outcome parameter. Persuing this parameter in isolation could be a futile exercise unless it is linked to the influencing variables such as patient sub groups, device, intervention, process and protocols. It is also important to understand that improvement in incidence of nosocomial infection does not necessarily mean improvement in quality unless it is linked to other parameters such as mortality outcome, length of stay, antibiotic consumption, cost implications etc. Mere reduction in the incidence of HAI rate without desired impact on the parameters mentioned earlier might not indicate qualitative improvement because such reduction is possible from change in the case mix.[5]

### 3. Factors Influencing Infection Rate

**3.1. Sub groups:** Overall infection loses its importance unless it is linked to patient sub groups such as; age, pre-morbid conditions, immunocompetence level. Therefore, infection rate could be different in a neonatal, pediatric, trauma, medical, surgical, burn and mixed ICUs. Infection rate will also be different in institution with predominance of a particular sub group of patients. Incidence in the institutions or units primarily managing cancer patients will be different than unit managing non cancer patients. Similarly result of a closed unit could be different as compared to an open unit.

**3.2. Device and Intervention Related:** Ventilator associated pneumonia, urinary catheter and invasive catheter or line related infection are device related parameters. Percentage of patients being maintained on different devices will influence the infection rate.

**3.3. Processes and Protocols:** Compliance to protocols, processes, guidelines, work instructions are also important determinants of infection rate. Process and protocol could be linked to antibiotic usage, investigations done, implementations of different treatment bundles, nursing care (line care, tracheostomy care), and hand hygiene.[6] Uniformity of practice through continuous training should be ensured and then compliance monitoring should be done.

**3.4. Infrastructure and Support Service:** Design of ICU, quality of water, laundry management, food handling, waste disposal, sterilization and other reprocessing and maintenance procedures, as well as microbiology support influence infection rate.

**3.5. Organizational, Human Resource and System Support:** Infection rate is also related to the organizational and human resource. Comparison of infection rate is possible if support level is similar in participating institution. Service provider related (nurse vs. patient, doctor vs. patient ratio) parameters should therefore be taken into consideration.

**3.6 Surveillance System:** Surveillance system available in the unit also makes a difference. Reliability of data is an important consideration, particularly if adequate staffing has not been ensured. Generating and stratifying voluminous data is labor intensive. Variability in reporting is possible in absence of electronic surveillance.[7,8]

### 4. Prioritization of parameter

Despite the availability of multiple parameters, it is practically not possible or logical to monitor all the possible parameters on long term basis. Prioritization of parameters therefore is essential to select those with maximum out put potential. Selection of limited few parameters, while the unit is getting quality-oriented, is also an alternative and easier approach. As the unit matures, need based addition and deletion can be done for the optional parameters but mandatory parameters should always be monitored. For example, if use of vancomycin is very limited in a particular unit then monitoring vancomycin resistant enteroccoci (VRE) is not logical on routine basis and can be taken as an optional parameter whereas line related infection could be a mandatory parameter. Certain key indicators should also be common and mandatory for inter institution, national or international comparison, accreditation and public reporting.

For prioritization, importance of parameters can be judged on a matrix where the Y axis represents determinants of importance and X axis represents a score of the determinants. Based on the overall score, prioritization can be done to select parameters. Example of such matrix is given below.

### Quality Indicators Matrix

**Table d32e4988:** 

Determinants	VAP	BSI	UTI	SSI
Ease of data collection				
Ease of definition				
Frequency of events				
Impact on mortality, morbidity, LOS				
Financial implications				
Ease of stratification				
Total score				

Score 1 to 5 (where 1= least important, 5= most important), VAP = ventilator associated pneumonia, BSI = Blood stream infection, UTI = Urinary tract infection, SSI = surgical site infection

### 5. Defining Parameter and Bench Marking

Whenever possible, parameter (numerator) should be linked to a denominator to make it more meaningful. Parameters used as denominator are: number of patients, bed occupancy days, number ventilated etc.[9]

Although international bench marks can be used for comparison of data, influence of geographical variation, nutritional and economical status etc. should be considered before comparing the result. It is therefore advisable to have national bench marks.

### 6. Common Quality Indicators Related to Infection:

**6.1. Device related Infection:** Ventilator associated pneumonia (VAP), Central line associated blood stream infection (BSI) and indwelling catheter related urinary tract infection (UTI) are commonly monitored parameters. Although VAP is being monitored very frequently wide variation in incidence is possible based on the diagnostic criteria used. Due to wide variation in surveillance definition, it is difficult to acquire, interpret and compare intra and inter institutional data.[10] Clinical and radiological diagnostic criteria are simplest. Quantitative and non quantitative culture of bronchial aspirate, quantitative culture of broncho-alveolar lavage (BAL) fluid and specimen collected by protected bronchial brush (PBS) are the other options. BAL and PBS are technically more challenging.

Despite claims and counterclaims, superiority of a particular technique could not be proved. Similar clinical outcomes and similar overall use of antibiotics had been recently claimed when nonquantitative culture of the bronchial aspirate and quantitative culture of BAL were compared for identification and subsequent management of VAP by the Canadian Critical Care Trials Group.[11] In order to bypass this controversy some authors advocate monitoring of risk factors which leads to VAP. This kind of monitoring is known as process oriented monitoring.[12] For example compliance monitoring to VAP prevention bundle can be taken as process quality parameter.[13]

Urinary tract infections are the second most common nosocomial infections in ICUs in Europe and the first in the United States.[14] Risk factors for bacteriuria should be taken into consideration such as: duration of catheterization, length of stay in the ICU, and female gender and drainage system. 2.96 cases of UTI per 100 admissions and 6.11per 1,000 device-days had been reported in medical ICU and 4.23 and 8.14 respectively had been reported in surgical ICU.[15]

Hospital-acquired bloodstream infection (BSI) alone has been estimated to be responsible for 26,250 deaths per year and ranks as the eighth leading cause of death in the United States. 3.55 cases of BSI per 100 admissions and 7.33per 1,000 device-days had been reported in medical ICU. Incidence varies depending upon the subset of patients managed in the ICU. In surgical ICU, 4.89 and 9.40 incidence of BSI per 100 patients and per 1000days had been reported respectively.[15]

**6.2. Infection from Specific Organism:** Infection due to *C. difficile* and methicillin-resistant *Staphylococcus aureus* extended spectrum beta lactamase producers (ESBL), Vancomycin resistant enerococci etc. could also be quality indicators. Infection caused by *C. difficile* and methicillin-resistant *Staphylococcus aureus* are being focused in US hospitals.[16]

**6.3. Antimicrobial use and Resistance Pattern:** Both antimicrobial use and drug resistance can be taken as quality indicators. For antimicrobial consumption, “Defined Daily Dose (DDD)” can be monitored. DDD of antimicrobial agent is calculated by dividing the total grams of the antimicrobial agent used in a hospital area by the number of grams in an average daily dose of the agent given to an adult patient.[17]

$\text{DDD per 1000 patient days}=\frac{\text{DDD of specific agent used}}{\text{Total no. of patient days}}\times 1000$

Similarly resistance rate can be calculated as:
$\frac{\text{Number of resistant isolates}}{\text{Number of isolates tested}}=\times 100$

### 7. Cost Effectiveness of Infection Control Program

Resources are not unlimited and therefore above mentioned parameters should be linked to the expenditure incurred for implementation of infection control programme. More than 30% reduction in infection rate had been claimed in the hospitals being monitored by National Nosocomial Infections Surveillance (NNIS) system which justifies expenditure on infection control.[17]

### 8. Summary

Nosocomial infection is a major concern due to multidimensional adverse influence it casts on the outcome, revenue, bed availability and patient satisfaction. Parameters should be selected considering the influence of HAI on patients and hospital management. Reduction in the incidence of infection should not be the only target; how this reduction is influencing long-term patient and hospital management should also be evaluated. Initially, very common parameters should be selected. Subsequently, based on the support and resource availability and need of the institution, focus can be shifted to other parameters.
